# Analysis of Human Thymopoiesis Reveals Rapid Postnatal Remodeling of Thymocytes Within the First 3 Months of Life

**DOI:** 10.1002/eji.70257

**Published:** 2026-08-18

**Authors:** Camille Kergaravat, Jean‐Marc Doisne, Clarisse Godard, Agata Cieslak, Mourad Amrane, David Vermijlen, Vahid Asnafi, Kamel Benlagha, James P. Di Santo, Emmanuel Clave, Antoine Toubert

**Affiliations:** ^1^ Institut de Recherche Saint Louis, INSERM UMR 1342 Université Paris Cité Paris France; ^2^ Innate Immunity Unit Institut Pasteur, INSERM U1223 Université Paris Cité Paris France; ^3^ Laboratoire d'Onco‐Hématologie, Hôpital Necker‐Enfants Malades Assistance Publique‐Hôpitaux de Paris (AP‐HP) Paris France; ^4^ Institut Necker Enfants Malades (INEM), INSERM U1151 Université Paris Cité, CNRS Paris France; ^5^ Service De Chirurgie Cardiovasculaire Hôpital Européen Georges Pompidou, AP‐HP Paris France; ^6^ Department of Pharmacotherapy and Pharmaceutics Université Libre de Bruxelles (ULB) Brussels Belgium; ^7^ Institute for Medical Immunology (IMI) Université Libre de Bruxelles (ULB) Gosselies Belgium; ^8^ ULB Center for Research in Immunology (U‐CRI) Brussels Belgium; ^9^ WELBIO Department WEL Research Institute Wavre Belgium; ^10^ Metabolism and Nutrition Research Group Louvain Drug Research Institute Université Catholique De Louvain Brussels Belgium

**Keywords:** perinatal immunity, postnatal immune development, thymopoiesis, unconventional T cells

## Abstract

The thymus generates a diverse and self‐tolerant T‐cell repertoire. Its development and output undergo profound changes from fetal to early postnatal life, yet the dynamics of this transition remain incompletely understood. Here, we performed a comprehensive analysis of human thymopoiesis across the perinatal (birth to <10 days), early postnatal (10 days–3 months), and homeostasis (>3 months) periods using high‐dimensional flow cytometry and signal joint T‐cell receptor excision circle (sjTREC) quantification. Within the perinatal phase, the thymus exhibits the highest intrathymic production, with elevated frequencies of CD4^+^ T cells and unconventional T cell (UTC), including regulatory T cells (Tregs). After 10 days of age, the frequency of double‐positive (DP) thymocytes increases and is associated with dynamic thymocyte phenotypic shifts that suggest enhanced progenitor commitment and proliferation, accompanied by reduced T‐cell receptor (TCR) signaling strength. Early postnatal UTCs (birth to 3 months) display a distinct phenotype compared with those generated after 3 months. We notably identified a postnatal wave of effector Vδ2^+^ γδ T cells emerging between 10 days and 3 months of age. These findings suggest a transition in human thymopoiesis from an early effector‐prone unconventional output to predominantly conventional T‐cell production after 3 months, highlighting a critical window in immune system establishment.

## Graphical abstract

Human thymopoiesis undergoes a dynamic postnatal transition, revealing maximal intrathymic sjTREC production at birth, shifts in thymocyte phenotype and composition, and a transient wave of effector Vδ2^+^ γδ T cells during early infancy.

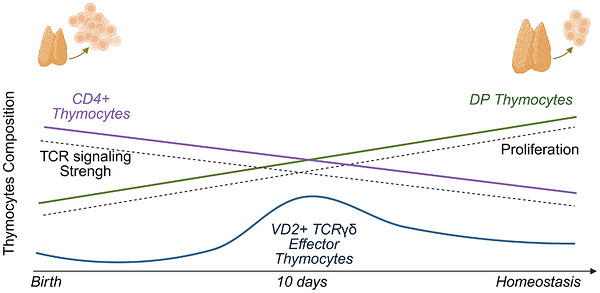

**Abbreviations**
APC: antigen‐presenting cells
cTEC: cortical thymic epithelial cells
DN: double negative
DP: double positive
iNKT: invariant natural killer T cell
ISP4: immature single‐positive CD4^+^ cells
MAIT: mucosal‐associated invariant T cell
MHC: major histocompatibility complex
mTEC: medullary thymic epithelial cells
pcw: postconception weeks
sjTREC: signal joint T‐cell receptor excision circle
SP: single positive
TCR: T‐cell receptor
TF: transcription factor
Tregs: regulatory T cells
UMAP: uniform manifold approximation and projection
UTCs: unconventional T cells

## 1 Introduction

The thymus is a primary lymphoid organ essential for generating a functional and self‐tolerant T‐cell repertoire that protects the body against infections and maintains homeostasis [1, 2]. Structurally, the thymus is organized in two main regions: the cortex, where early thymocyte development occurs, and the medulla, which establishes central tolerance. The cortical region is densely populated with immature lymphoid cells supported by cortical thymic epithelial cells (cTECs), whereas the medulla contains medullary thymic epithelial cells (mTECs) and thymic antigen‐presenting cells (APCs), including dendritic cells, B cells, plasmacytoid dendritic cells, and macrophages, which collectively shape the thymic microenvironment [3].

Thymopoiesis, the process of T‐cell generation, is highly regulated and proceeds through sequential stages defined by CD4 and CD8 expression: double‐negative (DN), double‐positive (DP), and single‐positive (SP) thymocytes. In humans, the DN population is further subdivided into DN1–DN3 stages on the basis of CD34, CD38, and CD1a expression, with full T‐lineage commitment marked by the acquisition of CD1a [4]. Following T‐lineage commitment, in humans, cells start to express the CD4 marker and thus become immature single‐positive CD4^+^ cells (ISP4). As thymocytes progress through these early stages, they undergo V(D)J recombination, rearranging variable (V), diversity (D), and joining (J) gene segments to generate their T‐cell receptors (TCRs) [4, 5]. This process gives rise to two major T‐cell lineages defined by their TCRs: γδ and αβ T cells. The γδ versus αβ lineage decision occurs at the DN stage, where successful rearrangement of the TCRγ and TCRδ loci promotes γδ T‐cell differentiation, whereas productive TCRβ rearrangement drives formation of the pre‐TCR and commitment to the αβ lineage [6]. These stages of thymopoiesis are highly dependent on Notch signaling [6, 7, 8, 9]. Following successful β‐selection, αβ‐committed thymocytes progress into the DP stage, where they undergo selection to ensure that self‐tolerant CD4^+^ or CD8^+^ SP T cells expressing functional αβ TCRs mature and exit the thymus. Positive selection occurs in the cortex, where thymocytes expressing TCRs capable of recognizing self‐major histocompatibility complex (MHC) molecules receive survival signals from cTECs, while non‐responsive cells undergo apoptosis. Selected thymocytes migrate to the medulla for negative selection, which is primarily mediated by mTECs and thymic APCs. Thymocytes with high‐affinity self‐reactive TCRs undergo apoptosis, preventing autoreactive T cells from entering the periphery [10]. However, some thymocytes experiencing strong TCR signals undergo agonist selection, diverting them into unconventional T‐cell (UTC) lineages rather than being deleted. These include invariant natural killer T (iNKT) cells, mucosal‐associated invariant T (MAIT) cells, γδ T‐cell subsets, CD8αα, and thymic regulatory T cells (Tregs) [11, 12, 13]. These cells acquire innate‐like effector programs in the thymus, with specialized functions: Tregs suppress autoreactive T cells, iNKT and MAIT cells provide rapid cytokine responses to microbial metabolites, γδ T cells contribute to early immune defense and tissue repair, and CD8αα T cells regulate mucosal immunity and epithelial tolerance [11, 12, 13].

Thymopoiesis begins early during fetal development; the thymus arises from the third pharyngeal pouch around 6 postconception weeks (pcw) and completes its organogenesis by 17 pcw [14, 15, 16]. Cortical and medullary regions appear around 8 pcw, providing the structural basis for thymocyte differentiation and selection. By 12 pcw, SP CD4^+^ and CD8^+^ thymocytes emerge. Fetal thymopoiesis is initiated by waves of hematopoietic progenitors from the fetal liver and bone marrow, producing mainly innate γδ T cells with invariant TCRs, followed later by conventional αβ T cells [15]. The fetal thymus generates abundant invariant and innate‐like subsets, a feature associated with the absence of DNA nucleotidylexotransferase (DNTT) in fetal hematopoietic stem cells [17, 18, 19].

The perinatal period, spanning the interval from late gestation through the first day after birth, represents a developmental window during which the immune system transitions from fetal to postnatal physiology. The shift from fetal to postnatal thymopoiesis is marked by both continuity and adaptation to the challenges of extrauterine life. In mouse models, perinatal thymic output has been associated with the generation of unique T‐cell subsets such as virtual memory CD8^+^ T cells and Tregs, which persist into adulthood and contribute to pathogen protection and the suppression of autoimmunity [20, 21, 22]. T cells generated during this period also display distinct functional properties, including hyposensitive TCR signaling and Th2‐biased responses, highlighting key differences between perinatal and later postnatal thymopoiesis when homeostasis is reached [23]. Much less is known about this period in humans. At birth, the human thymus produces high levels of conventional αβ T cells alongside innate‐like subsets. After birth the thymus produces mainly conventional αβ T cells [15]. Thymic tissue homeostasis is defined by thymus growth arrest and has been reported to be reached around 3 months after birth in humans [24].

Thymus production is influenced by several factors. Sex has been reported has a major factor influencing thymic output in periphery [25], likely through its influence on thymic organ architecture [26]. Aging also influences strongly thymic function due to organ involution. Involution occurs in multiple phases: rapid reduction in thymic mass after 1 year of life, followed by a slower decline during adulthood, with an estimated loss of ∼3% per year early in life, decreasing to <1% per year in later adulthood [27]. Thymic involution is associated with size reduction, disorganization of cortical and medullary architecture, and decline in T cell production. Despite this, the thymus continues to supply at a reduced pace new naïve T cells during childhood and adulthood, supporting a diverse T‐cell repertoire [25, 28]. With aging, reduced thymic output, along with accumulation of memory and senescent T cells, contributes to impaired immune responsiveness and increased susceptibility to infections, cancer, and autoimmunity [29, 30]. Thymus health has been recently recognized as a predictor of lifelong well‐being and immunotherapy effectiveness [31, 49].

Understanding thymocyte development—from neonatal expansion to postnatal homeostasis—is essential for uncovering how early‐life immune competence is established and how the foundations of immune system are laid. In this study, we employ high‐dimensional flow cytometry and signal joint TCR excision circle (sjTREC) quantification to profile thymic output and cellular composition from the perinatal period (birth to <10 days) through early postnatal period (10 days–3 months) until homeostasis is reached (>3 months), a developmental window that has been largely understudied in humans.

## 2 Results

### 2.1 Thymocyte Composition in Humans

**FIGURE 1:**
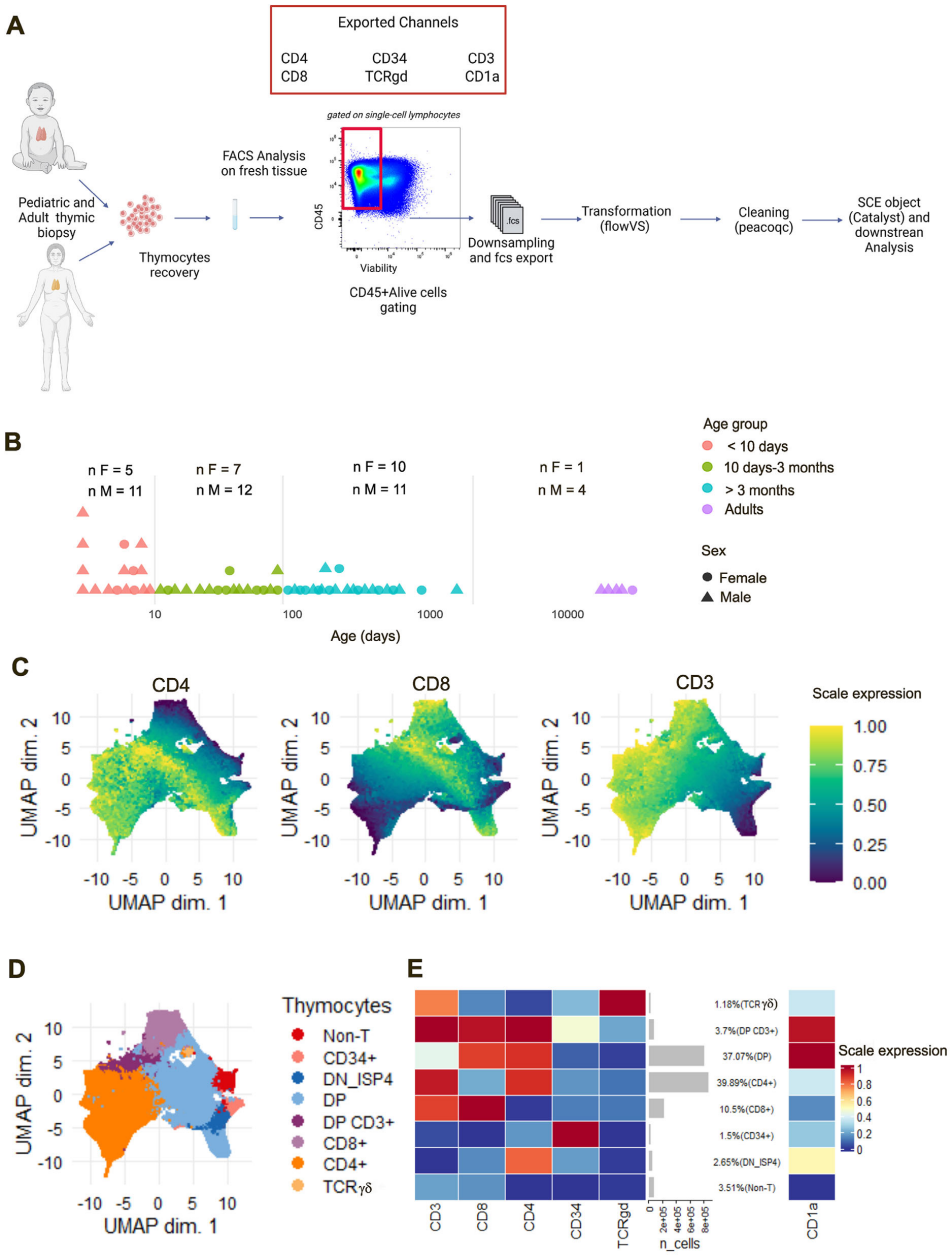
Human thymocyte composition in thymic tissue. (A) Schematic representation of the experimental design, including the gating strategy, selection of flow cytometry panel markers, and the unsupervised analysis pipeline used for FCS file processing and integration to characterize thymocyte populations. (B) Summary of thymus samples included in the study, stratified by age and sex. Flow cytometry analyses were performed on samples from a total of 56 pediatric donors and 5 adult donors. (C) UMAP projection of human thymocytes depicting scaled expression levels of CD4, CD8, and CD3. (D) UMAP projection of human thymocytes colored according to annotated clusters. (E) Heatmaps showing scaled median marker expression across clusters. Markers used for cluster annotation are displayed in the left heatmap, whereas CD1a expression within each cluster is shown in the right heatmap. Gray bars indicate the number of cells per cluster and their relative frequency within the total thymocyte population. DN, double negative; DP, double positive; SCE, SingleCellExperiment; TCR, T‐cell receptor; UMAP, uniform manifold approximation and projection.

To define the baseline landscape of thymocyte populations, we analyzed fresh thymic tissue recovered during cardiac surgery from children and adults. As thymocytes are highly fragile, analysis was performed immediately after isolation to maximize cell integrity. Thymocytes were recovered from the tissue, and the main thymocyte populations were assessed by spectral flow cytometry (Figure 1A). Samples were collected from both male and female age‐matched donors, with equivalent representation (Figure 1B). We focused our analysis on pediatrics samples on three age groups: from birth to 10 days old (perinatal; <10 days), between 10 days and 3 months (early postnatal; 10 days–3 months), and older than 3 months up to 4 years old (homeostasis; >3 months). These groupings were chosen to reflect established phases of postnatal immune maturation and to uncover perinatal to homeostasis dynamics of thymopoiesis in humans. They are supported by evidence in humans showing convergence of immune trajectories within 3 months of life [32], peak thymic growth, and thymic output's dynamics within the first month of life and stabilization around 3–4 months [24, 33]. Fifty‐six pediatric samples were included in the analysis, enabling robust evaluation of population dynamics within these groups. Samples were stained to identify major thymocyte subsets (Figure 1A), and an unbiased single‐cell analysis was performed. Live CD45^+^ cells were gated, downsampled, transformed, and quality‐controlled before integration into a single‐cell experiment object (Figure S1A). The object was subjected to the FlowSOM algorithm to group phenotypically similar cells, which were subsequently visualized using uniform manifold approximation and projection (UMAP) embedding. Using CD3, CD4, and CD8 markers, we broadly identified the main thymocyte subsets: DN, DP, and SP (CD4 or CD8) cells (Figure 1C). We further refined this annotation using all exported markers, identifying clusters that trace T‐cell differentiation from CD34^+^ thymocytes to SP cells (Figure 1D,E). All cell subpopulations were recovered in all samples included in the analysis (Figure S1B). Immature thymocytes accounted for <5% of total cells, including CD34^+^ thymocytes (1.6%) and a DN–ISP4 cluster (3.0%). The DN–ISP4 cluster contained cells with phenotypic features spanning both DN and immature single‐positive CD4 (ISP4) thymocytes and was, therefore, annotated as DN–ISP4. DP cells represented approximately 41% of total thymocytes, followed by CD4 SP cells (Figure 1E). These results slightly differ from previously reported thymocyte proportions [34, 35]. Our staining strategy included intracellular staining, which required fixation of thymocyte suspensions prior to acquisition. As DP thymocytes are particularly sensitive to stress and manipulation, fixation resulted in a reduced representation of DP cells and a relative increase in CD4 SP thymocytes. Analysis of the same thymic sample, freshly stained but unfixed, showed DP and CD4 SP frequencies consistent with previous reports in the literature (Figure S2A,B). Importantly, frequencies obtained before and after fixation were correlated, and the impact of fixation was comparable across age groups (Figure S2C–F). Frequencies derived from unsupervised clustering were highly correlated with those obtained by conventional manual gating (Figure S2G), supporting the robustness of our analytical approach.

Therefore, this analysis identifies the major thymocyte subsets and provides a reference for subsequent age and sex‐related comparisons.

### 2.2 Influence of Age and Sex on Thymocyte Populations

We next assessed how thymocyte composition and thymic output vary with age and sex during the transition from the perinatal period (<10 days) through early postnatal periods (10 days–3 months) until homeostasis (>3 months) in pediatric donors.

**FIGURE 2:**
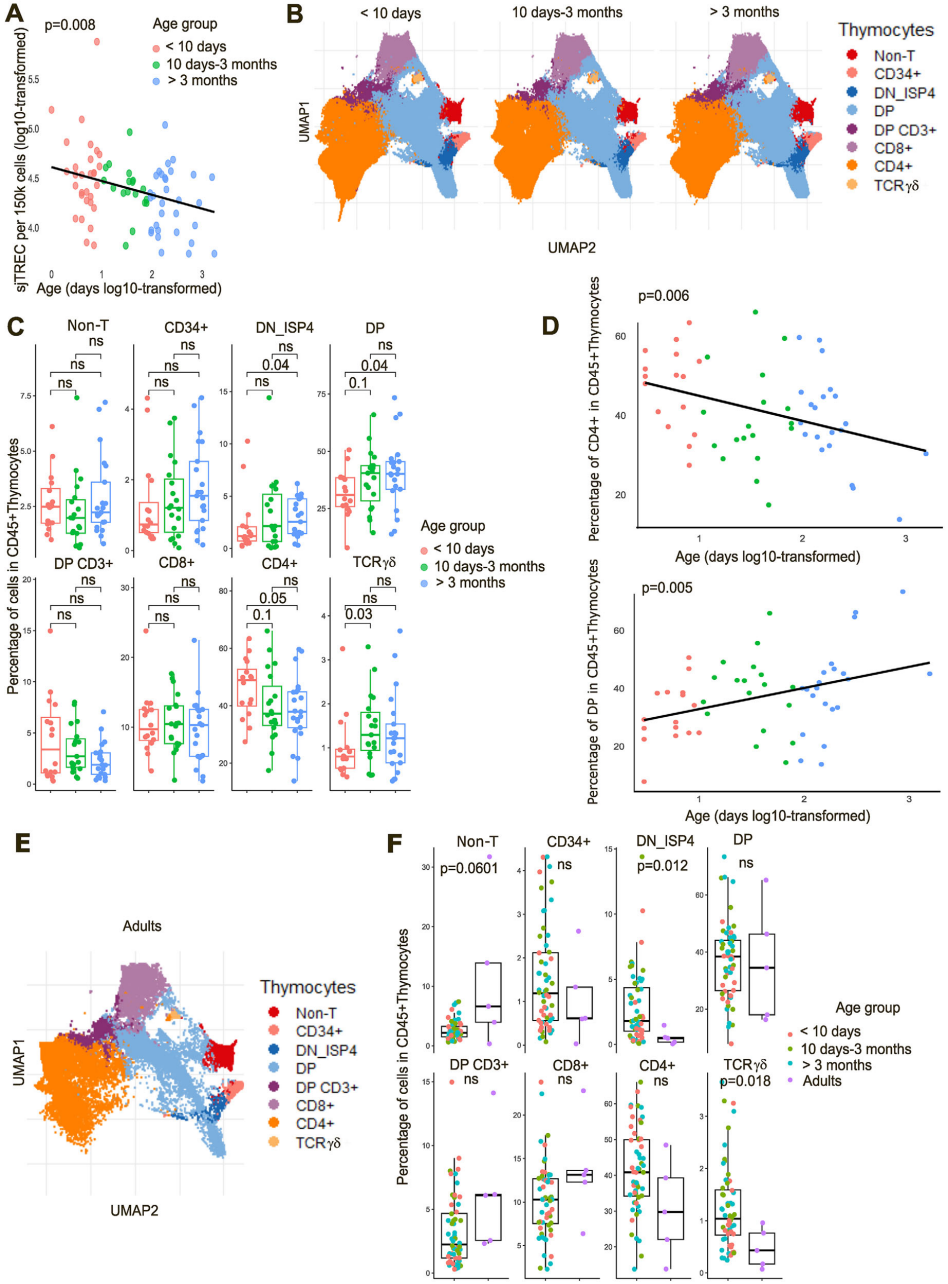
Age‐related changes in thymocyte composition from the perinatal period to homeostasis. Sample numbers and performed analysis are detailed in Figure S8 and Table S5. (A) sjTREC levels according to age. Linear regression analysis of log10‐transformed sjTREC copies per 150,000 cells as a function of log10‐transformed age (<10 days, *n* = 29; 10 days–3 months, *n* = 18; >3 months, *n* = 31). The *p* value corresponds to the significance of the regression slope. (B) UMAP projection of pediatric human thymocytes stratified according to age group. (C) Boxplots showing the frequency of each cluster within the total pediatric thymocyte population across age groups (<10 days, *n* = 16; 10 days–3 months, *n* = 19; >3 months, *n* = 21). *p* values correspond to pairwise comparisons between age groups and were calculated using two‐sided Wilcoxon rank‐sum tests. (D) Linear regression analysis of CD4^+^ (top) and DP (double‐positive; bottom) thymocyte frequencies within the total thymocyte population as a function of log10‐transformed age. CD4^+^ thymocytes were enriched during the first 10 days of life, whereas DP thymocyte frequencies increased with age (<10 days, *n* = 16; 10 days–3 months, *n* = 19; >3 months, *n* = 21). *p* values correspond to the significance of the regression slope. (E) UMAP projection of adult human thymocytes colored according to annotated clusters. (F) Boxplots comparing the frequency of each cluster within the total thymocyte population between pediatric (*n* = 56) and adult (*n* = 5) samples. *p* values were calculated using the Wilcoxon rank‐sum test. DN, double negative; DP, double positive; sjTREC, signal joint T‐cell receptor excision circle; TCR, T‐cell receptor; UMAP, uniform manifold approximation and projection.

First, we evaluated sjTREC levels according to age. We observed that sjTREC levels decline with age (Figure 2A), especially after 3 months of life compared to the first week of life (Figure S3A). These results suggest a reduction in thymic production between birth and homeostasis.

We then compared thymic subpopulation frequencies to determine if the decline in thymic production was associated with any population shift (Figure 2B,C). In UMAP visualization by age group, no novel population appeared or disappeared with age. However, we observed clear shifts in both CD4 and DP subpopulations: CD4 frequencies decreased with age, whereas DP frequencies increased (Figure 2C,D). These results were confirmed in the unfixed samples (Figure S3B) and are consistent with previously published data [35]. Other populations did not appear to be affected by age in a linear manner (Figure S3C). There were no significant sex‐associated differences in thymocyte subsets. We observed an increase in non‐T‐cell cluster frequency in males (Figure S3D).

We then investigated whether thymocyte generation is sustained throughout life by analyzing adult thymus samples from donors aged 48–81 years. Adult thymi contained thymocyte populations from CD34^+^ to SP cells, organized into clusters analogous to pediatric samples (Figure 2E), indicating ongoing thymopoiesis in older individuals. Cluster frequency analysis revealed age‐related changes: reductions in DN–ISP4 and TCRγδ^+^ populations, with a trend toward increased CD8^+^ and decreased CD4^+^ representation, suggesting shifts in the CD4/CD8 ratio (Figure 2F, Figure S4). The reduction in progenitors is consistent with the decreased capacity of the aged thymus to sustain thymopoiesis as actively as in children and aligns with reports of reduced T‐lineage potential in CD34^+^ cells with age [36]. The decrease in CD4^+^ frequency could reflect the well‐documented reduction in thymic output in the elderly, whereas the relative increase in CD8^+^ cells has also been observed in humans over 40 years old compared to younger individuals [36]. Non‐T‐cell populations also trend to increase with age (Figure 2F, Figure S4), consistent with reports of increase in B cells, myeloid cells, and NK/ILC representation [36, 37].

Overall, this quantitative analysis shows dynamic shifts of thymopoiesis in specific subsets from perinatal through early postnatal period until homeostasis is reached.

### 2.3 High‐Dimensional Profiling of Postnatal Thymocyte Population Dynamics

**FIGURE 3:**
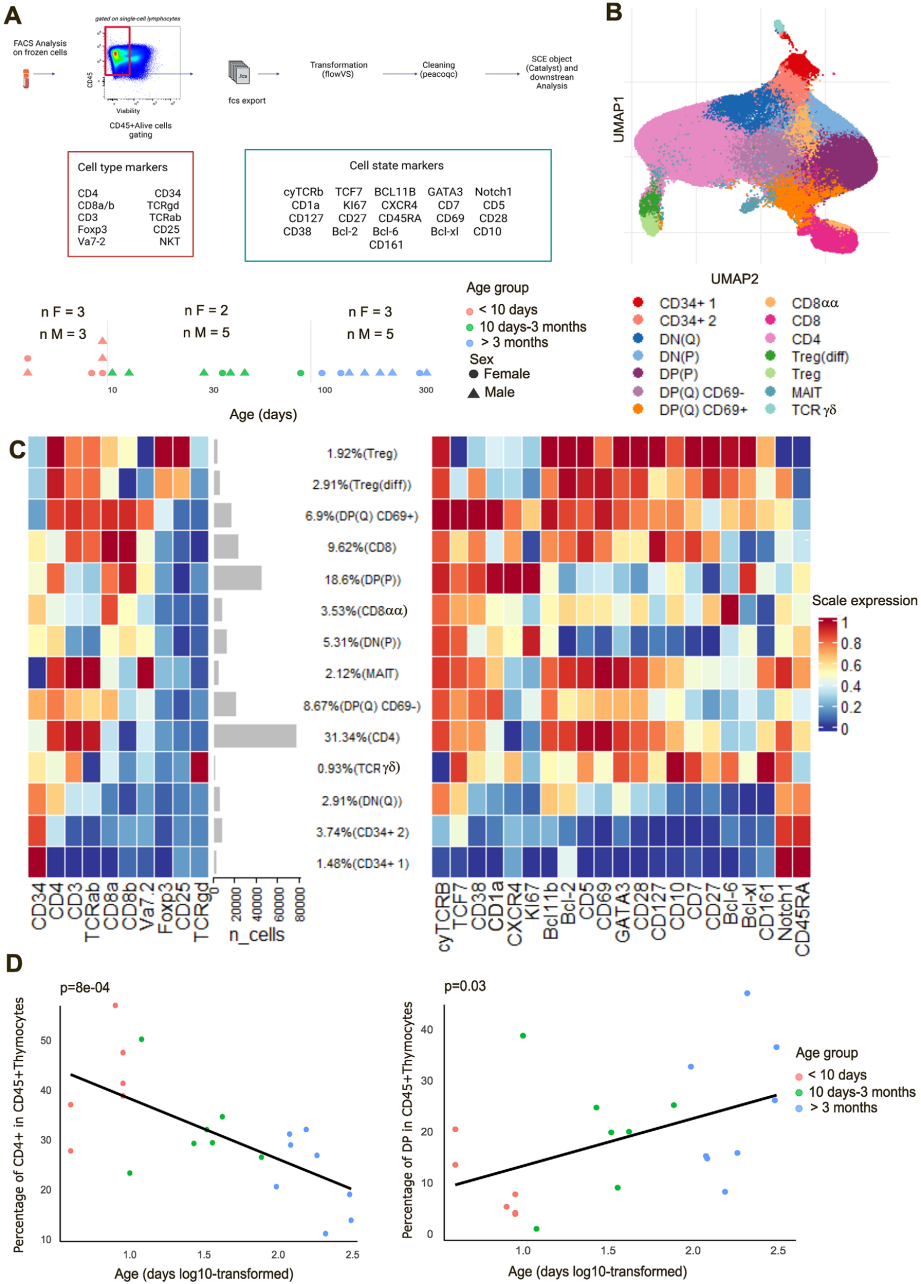
Refined mapping of postnatal thymocyte developmental dynamics. (A) Schematic representation of the experimental design, including the gating strategy and the unsupervised analysis pipeline used for FCS file processing and integration to characterize pediatric thymocyte populations from biobanked frozen samples. A summary of thymus samples included in the study, stratified by age and sex, is shown. Flow cytometry analyses were performed on samples from a total of 21 donors. Sample numbers and performed analysis are detailed in Figure S8 and Table S5. (B) UMAP projection of human thymocytes colored according to annotated clusters. (C) Heatmaps showing scaled median marker expression across clusters. Markers used for cluster annotation are displayed in the left heatmap, whereas markers characterizing cell states within each cluster are shown in the right heatmap. Gray bars indicate the number of cells per cluster and their relative frequency within the total thymocyte population. (D) Linear regression analysis of CD4^+^ (left) and DP (double‐positive; right) thymocyte frequencies within the total thymocyte population as a function of log10‐transformed age. Analysis of frozen thymocytes confirmed the enrichment of CD4^+^ thymocytes during the first 10 days of life and the progressive increase in DP thymocyte frequencies with age (<10 days, *n* = 6; 10 days–3 months, *n* = 7; >3 months, *n* = 8). *p* values correspond to the significance of the regression slope. DN, double negative; DP, double positive; MAIT, mucosal‐associated invariant T cell; TCR, T‐cell receptor; Treg, regulatory T cell; UMAP, uniform manifold approximation and projection.

To validate the robustness of our findings and to enable a deeper characterization, we performed high‐dimensional phenotypic profiling on biobanked frozen pediatric thymocyte samples using a 35‐marker panel (Figure 3A). This panel was designed to identify conventional as well as unconventional populations (MAIT, iNKT, CD8αα, and Tregs) and to have a deep cell‐state evaluation (Figure 3A).

This analysis was performed on 21 thymic pediatric samples, evenly distributed across age groups, using a workflow similar to fresh samples (Figure 3A). This approach enabled the identification of different immature subpopulations (CD34^+^1, CD34^+^2, DN(Q), DN(P)—with Q for Quiescent and P for Proliferative), various DP populations (DP(Q) CD69^−^, DP(P), DP(Q) CD69^+^), unconventional populations (CD8αα, MAIT, TCRγδ, Treg(diff), Treg), and SP cells (CD4, CD8), thereby almost recovering the full spectrum of known thymocyte populations (Figure 3B,C). Treg(diff) represents an intermediate population of cells committed to the Treg differentiation pathway. iNKT were barely detectable due to their poor representation in human thymus, precluding a consistent analysis of this population (Figure S5A).

Markers used to define each population are shown on the left heatmap (Figure 3C). Additionally, using additional included markers represented on the right heatmap, we could finely discriminate different thymocyte subsets (Figure 3C). Two CD34^+^ subpopulations were identified: CD34^+^1 cells, which do not yet express T‐lineage transcription factors (TF) and exhibit Bcl‐2 expression, and CD34^+^2 cells, which show mild expression of T‐lineage TFs. The DN(Q) population represents the first immature subset to express high levels of T‐lineage TFs (TCF7, GATA‐3, Bcl11b) and TCRβ (cyTCRβ). These three subsets represent the most immature subpopulations as illustrated by the high Notch1 expression and no CD1a expression within these subsets. Strong T‐cell commitment is evident from the DN(P) cluster, marked by high CD1a expression. Thymocyte proliferation, assessed by Ki‐67 expression monitoring, was detected in DN(P), DP(P), and DP(Q) CD69^+^ clusters. Apoptosis‐related markers (Bcl‐2, Bcl‐xl) further refined the characterization of cell states: Bcl‐2 was strongly expressed in all non‐proliferating thymocyte populations, whereas Bcl‐xl was expressed in both agonist‐selected populations Treg, TCR γδ and proliferating cells. Bcl‐6 expression was enriched in agonist‐selected subsets. These proliferation and survival patterns followed TCR signaling events in DP(P) and DP(Q) CD69^+^ clusters, monitored through CD5, CD28, and CD69 expression. Population maturity was further assessed using CD127 and CD45RA, markers that are associated with a mature naïve phenotype. Importantly, these populations were consistently recovered across all samples (Figure S5B). Moreover, we were able to confirm the CD4 and DP population shifts in frequency according to age (Figure 3D).

Some samples were included in both analyses, enabling evaluation of thawing effect on thymocyte population representation. We observed differences in population distributions following thawing, including an increase in immature populations and a relative reduction in DP and CD4^+^ cell frequencies (Figure S5C). This indicates that sample freezing influences cellular distribution and should be considered when interpreting downstream analyses. However, changes were consistent across age groups and are therefore unlikely to confound analyses of age‐related dynamics. No new age‐associated population shift occurred specifically in frozen samples (Figure S5D), and marker expression was equally distributed across samples (Figure S6).

These data recapitulate population dynamics observed in fresh thymic tissue, allowing deeper investigation of thymocyte subpopulation dynamics with age.

### 2.4 Age‐Associated Changes in Marker Expression Within Conventional Thymocyte Subsets

**FIGURE 4:**
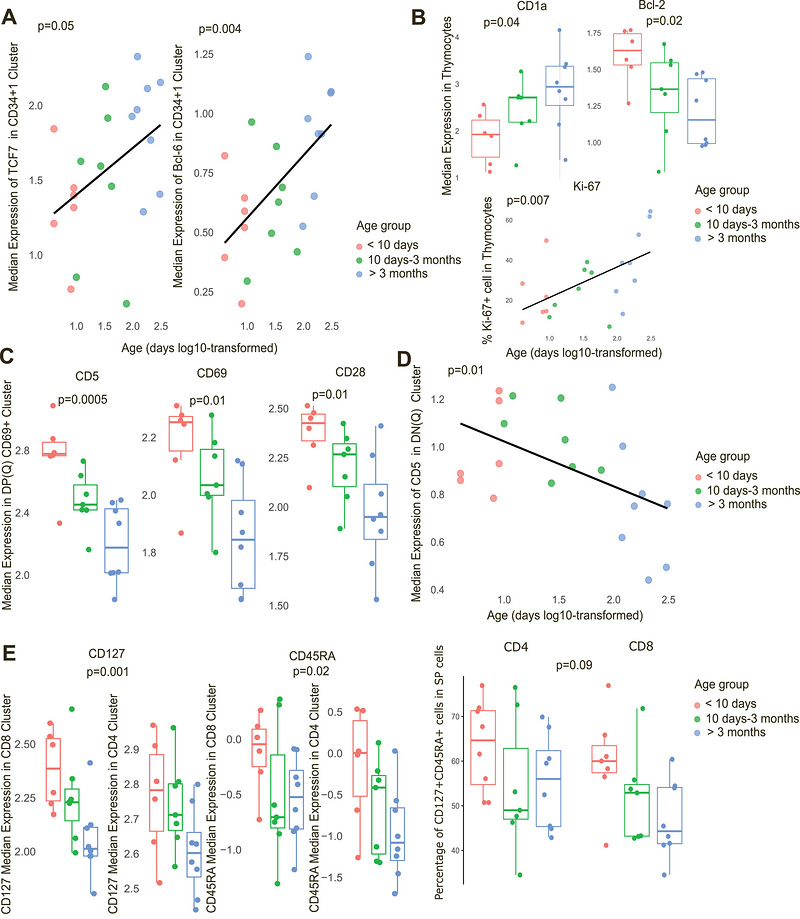
Age‐associated modulation of marker expression within conventional pediatric thymocyte subsets. (A) Linear regression analysis of TCF7 (left) and Bcl‐6 (right) median expression as a function of log10‐transformed age within the CD34^+^1 cluster (<10 days, *n* = 6; 10 days–3 months, *n* = 7; >3 months, *n* = 8). *p* values correspond to the significance of the regression slope. (B) Boxplots showing median CD1a and Bcl‐2 expression across age groups (top) and linear regression analysis of Ki‐67^+^ thymocytes frequencies as a function of log10‐transformed age (bottom; <10 days, *n* = 6; 10 days–3 months, *n* = 7; >3 months, *n* = 8). *p* values for CD1a and Bcl‐2 were calculated using the Kruskal–Wallis test. *p* value for Ki‐67 corresponds to the significance of the regression slope. (C) Boxplots showing median CD5, CD69, and CD28 expression within the DP(Q) CD69^+^ cluster across age groups, revealing a progressive reduction in markers associated with T‐cell receptor signaling with increasing age (<10 days, *n* = 6; 10 days–3 months, *n* = 7; >3 months, *n* = 8). *p* values were calculated using the Kruskal–Wallis test. (D) Linear regression analysis of CD5 median expression as a function of log10‐transformed age within the DN(Q) cluster (<10 days, *n* = 6; 10 days–3 months, *n* = 7; >3 months, *n* = 8). The *p* value corresponds to the significance of the regression slope. (E) Left, boxplots showing median CD127 and CD45RA expression within CD4 and CD8 single‐positive (SP) thymocyte subsets across age groups. Right, boxplots showing the frequency of CD127^+^CD45RA^+^ cells within CD4 and CD8 SP thymocyte subsets (<10 days, *n* = 6; 10 days–3 months, *n* = 7; >3 months, *n* = 8). *p* values for median fluorescence intensity (MFI) measurements were calculated using two‐way ANOVA. *p* values for CD127^+^CD45RA^+^ cell frequencies were calculated using a mixed‐effects model with age group and SP subset as fixed effects. Shown *p* values correspond to age group effect. DP, double positive.

We next examined the expression of key surface and intracellular markers across conventional thymocyte subsets. In the most immature population (CD34^+^1), both TCF7 and Bcl‐6 expressions increased with age (Figure 4A). TCF7 is essential for initiating the core T‐cell transcriptional program, whereas Bcl‐6 contributes to cell survival and cellular quiescence, suggesting age‐related modulation of early thymopoiesis. We also found in total thymocytes that CD1a expression and Ki‐67 positive cells increased with age, whereas Bcl‐2 expression decreased (Figure 4B). CD1a marks T‐lineage commitment, Ki‐67 reflects proliferation, and Bcl‐2 promotes survival. Together, these changes indicate increasing commitment, proliferative capacity, and less resistance to apoptosis among developing thymocytes with age in young children. This pattern suggests a shift in the regulation of thymopoiesis from perinatal period to homeostasis that may originate from the remodeling of the most immature progenitors.

In the DP(Q) CD69^+^ cluster, representing post‐positive selection DP cells, markers associated with TCR signaling strength (CD5, CD69, CD28) decreased with age, suggesting reduced TCR signaling after the first 10 days after birth (Figure 4C). Similarly, DN(Q) cells showed reduced CD5 expression, indicative of diminished β‐selection signaling strength (Figure 4D). These findings align with previous observations in mice and humans showing that perinatal thymocytes exhibit higher TCR signaling levels with higher CD5 and Nur77 expression compared to those generated later in life [20].

In SP clusters, we observed an age‐associated reduction in the median expression of CD127 and CD45RA (Figure 4E). This finding was modestly supported by analysis of cell frequencies, which showed a corresponding trend of decrease with age in the percentage of CD127^+^CD45RA^+^ cells within SP clusters (Figure 4E).

Overall, these phenotypic data suggest a coordinated maturation of thymopoiesis within a very short time frame from the perinatal (<10 days) through early postnatal period (10 days–3 months) into thymic homeostasis (>3 months), characterized by an increased progenitor T‐lineage commitment and proliferation together with a reduced TCR signaling strength, having a phenotypic impact on the T‐cell produced.

### 2.5 Specific Age‐Related Patterns in Unconventional Thymocyte Subsets

In mice, UTCs generated during the perinatal period are unique and have lasting consequences for immune homeostasis [20, 21, 22]. To evaluate if similar dynamics exist in humans, we next analyzed human UTC in our samples according to age.

**FIGURE 5:**
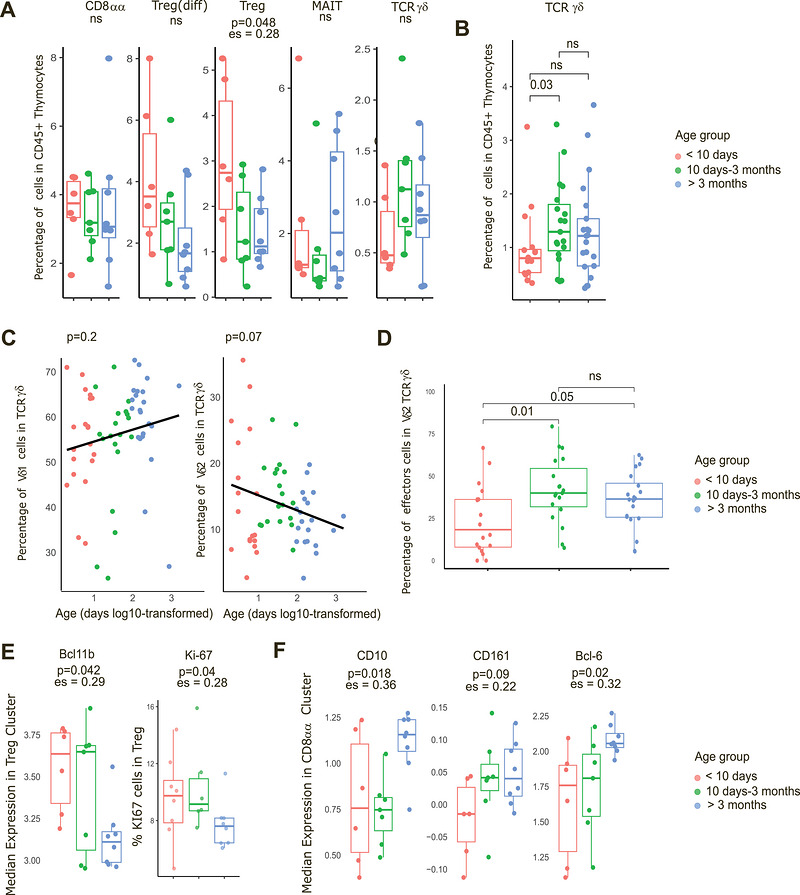
Age‐related changes within unconventional pediatric thymocyte subsets. (A) Boxplots showing the frequency of unconventional thymocyte populations across age groups (<10 days, *n* = 6; 10 days–3 months, *n* = 7; >3 months, *n* = 8). *p* values were calculated using one‐way ANOVA. (B) Boxplots showing TCRγδ thymocyte frequencies in fresh thymus samples across age groups (<10 days, *n* = 16; 10 days–3 months, *n* = 19; >3 months, *n* = 21). *p* values correspond to pairwise comparisons between age groups and were calculated using two‐sided Wilcoxon rank‐sum tests. (C) Linear regression analysis of Vδ1^+^ TCRγδ (left) and Vδ2^+^ TCRγδ (right) cell frequencies within TCRγδ as a function of log10‐transformed age (<10 days, *n* = 18; 10 days–3 months, *n* = 18; >3 months, *n* = 21). *p* values correspond to the significance of the regression slope. (D) Boxplots showing the frequency of effector TCRγδ cells within the Vδ2^+^ TCRγδ compartment across age groups, revealing a postnatal wave of effector TCRγδ cells between 10 days and 3 months of age (<10 days, *n* = 18; 10 days–3 months, *n* = 18; >3 months, *n* = 21). *p* values correspond to pairwise comparisons between age groups and were calculated using two‐sided Wilcoxon rank‐sum tests. (E) Boxplots showing Bcl11b median expression (left) and the frequency of Ki‐67^+^ cells (right) within the Treg cluster across age groups (<10 days, *n* = 6; 10 days–3 months, *n* = 7; >3 months, *n* = 8). *p* values were calculated using one‐way ANOVA. (F) Boxplots showing CD10, CD161, and Bcl‐6 median expression within the CD8αα cluster across age groups (<10 days, *n* = 6; 10 days–3 months, *n* = 7; >3 months, *n* = 8). *p* values were calculated using one‐way ANOVA. MAIT, mucosal‐associated invariant T cell; TCR, T‐cell receptor; Treg, regulatory T cell.

In human thymus, MAIT cell frequencies remained stable with age, whereas CD8αα and Treg (diff) populations showed a trend toward reduction (Figure 5A) in line with publications that suggest their production during early life [15, 38]. Treg frequencies decreased significantly with age, although the statistical significance was modest (*p* = 0.048). Nevertheless, the observed effect size (0.28) indicates that age accounted for a substantial proportion of the variation in Treg frequency (Figure 5A).

In contrast, TCRγδ^+^ cell frequencies seem to increase between 10 days and 3 months, followed by a trend toward reduction after 3 months. This dynamic was confirmed in fresh samples (Figure 5B), highlighting unique kinetics of the γδ T‐cell population. As γδ T cells can be classified based on their variable chain usage, with Vδ1 usage being linked to adaptive γδ T‐cell and Vδ2 usage being linked to both adaptive γδ T‐cell and innate effector γδ T cell [17, 39], we next assessed TCRδ variable (Vδ) chain usage and effector marker expression within this population. The full list of markers is provided in Table S1, and the gating strategy used to identify γδ T‐cell subsets is shown in Figure S7A. Within these samples, Vδ1 γδ cells showed a trend toward increase, whereas Vδ2 γδ cells trended to decrease upon aging, but no significant difference in variable chain usage was associated with the γδ frequency increase (Figure 5C, Figure S7B). Interestingly, the overall increase in γδ T frequency between 10 days and 3 months was associated with a higher frequency of effector cells defined by the expression of CCR6^+^CD161^+^CD26^+^ within the Vδ2 subset (Figure 5D) [40, 41].

Analysis of marker expression within Tregs cluster revealed decreased percentage of Ki‐67 positive cells and expression of Bcl11b after 3 months (Figure 5E). Reduced Ki‐67 positive cells confirm previous findings in mice that perinatal Tregs display enhanced proliferative capacity, which we show to be conserved in humans [22, 42]. The Treg(diff) cluster is defined by reduced expression of CD25 and FoxP3 and includes Treg precursors. A similar reduction in Bcl11b expression was observed in the Treg(diff) cluster (Figure S7B), suggesting that decreased Bcl11b expression with age is associated with the Treg lineage. We did not observe changes in CD5 expression within this population with age (Figure S7C), despite recent reports implicating CD5 in the induction of Treg identity and function [43]. In CD8αα cells, expression of CD10, CD161, and Bcl‐6 increased with age (Figure 5F), suggesting that although the overall production of these cells appears to decrease over time, those generated later in life exhibit a more mature phenotype, as these markers are characteristic of this agonist‐selected T‐cell subpopulation [38]. Marker‐expression differences observed in these UTC populations reached small statistical significance. These findings should therefore be interpreted cautiously, particularly given the limited sample size. However, the corresponding effect sizes (Figure 5 and Table S4) indicate that some of these differences may be biologically meaningful and warrant validation in larger cohorts.

Overall, these findings reveal specific dynamics and remodeling of unconventional thymocyte subset generation from the perinatal (<10 days) through early postnatal period (10 days–3 months) into thymic homeostasis (>3 months), likely reflecting changes in thymic crosstalk and V(D)J recombination with age.

## 3 Discussion

In this study, we provide a comprehensive characterization of human thymopoiesis across the perinatal (<10 days), early postnatal (10 days–3 months), and homeostasis (>3 months) periods and report how thymic output and T‐cell differentiation evolve in early life. Age groups in this study were defined to facilitate visualization and interpretation of changes occurring during early postnatal thymopoiesis maturation and do not imply strict biological boundaries as main findings were derived from analysis using age as a continuous variable. The perinatal period is a critical period for the immune system setup and has not been studied in‐depth in humans, as most flow cytometry studies have been conducted in smaller cohorts and older donors [34, 35]. Through high‐dimensional phenotyping of thymic samples, we identified marked remodeling of both conventional and unconventional thymocyte populations as the thymus transitions from the perinatal window through early postnatal into thymic homeostasis. Our analysis suggests that the perinatal period (<10 days) is defined by maximal thymic production and enriched frequencies of CD4^+^ and UTC. This age‐associated decline in thymic production after perinatal period is supported by decreased sjTREC levels; however, this interpretation should be made with caution because sjTREC abundance may also be influenced by changes in thymocyte proliferation. Our phenotypic analyses argue that proliferation is unlikely to be the sole driver of the observed decrease, as the major proliferative events occur predominantly in developmental stages preceding sjTREC generation. Nevertheless, we cannot exclude the contribution of proliferation to bias sjTREC levels. Future studies using complementary approaches to assess thymic production will be required to confirm this finding. We also found evidence implying that UTC generated during early postnatal period (from birth to 3‐month‐old) are phenotypically distinct from their later postnatal (>3 months) counterpart, including Tregs with an increased proliferative capacity and CD8αα cells with reduced lineage‐marker expression.

We observed a previously undocumented increase in effector Vδ2 γδ T cells between 10 days and 3 months, consistent with the possibility that a postnatal wave of effector γδ T‐cell production occurs very early after birth [40]. A main fraction of effector Vδ2 γδ thymocytes are phosphoantigen‐reactive Vγ9Vδ2 T cells [19, 39]. One possible explanation for the observed rise in effector γδ Vδ2 thymocytes after birth is the exposure to microbiome‐derived phosphoantigens traveling to the thymus, similarly to mechanisms described for MAIT cells [44, 45], and may contribute to the early expansion in blood Vγ9Vδ2 T cells early after birth [46, 47]. However, alternative non‐mutually exclusive explanations should also be considered, including changes in proliferation, tissue egress dynamics, or delayed maturation. Further functional studies will be required to distinguish between these possibilities. Effector Vγ9Vδ2 T cells have been shown to be involved in responses toward *Toxoplasma gondii*, malaria infections, and sepsis in human neonates [48]; thus, investigation of factors driving their expansion is of interest.

As thymopoiesis progresses beyond the perinatal period (>10 days), we report an increase in DP thymocyte frequency and phenotypes indicative of enhanced progenitor commitment, reduced TCR signaling strength, and changes in phenotype of SP cells. As the CD127^+^CD45RA^+^ phenotype corresponds to terminally differentiated naïve mature SP thymocytes, the age‐associated decrease in these markers suggests that SP thymocytes generated 10 days after birth display a less terminally differentiated phenotype compared to those produced during the perinatal period (<10 days).

These data suggest a coordinated maturation of thymopoiesis within a relatively short time frame from an apparently effector‐biased perinatal period into thymic homeostasis predominantly associated with conventional T‐cell production. These results align with mouse data, which show that T cells generated during perinatal period display distinct functional properties, including hyposensitive TCR signaling and increased effector properties [20, 23]. Furthermore, it corroborates the postnatal shifts in UTC described in murine thymus, providing evidence that similar developmental patterns may occur in the human thymus. However, given the limited sample size available for some age groups, these observations should be considered preliminary and will require validation in larger independent cohorts.

Sex‐stratified analyses suggest that early thymopoiesis is largely sex‐independent in our cohort, as we did not observe significant sex‐associated differences in thymocyte composition. This contrasts with prior reports describing sex‐related differences in thymic compartments in 2–3‐month‐old children [26]. This lack of detectable differences may reflect limited power to resolve subtle effects with flow cytometry or stage‐specific sex differences not captured within the sampled developmental window. Further studies in larger cohorts using additional marker inclusion will be required to fully delineate potential sex‐associated influences on thymocyte composition.

Finally, analysis of adult thymi demonstrates that, as already reported, although there is a decline in progenitors, CD4^+^, and γδ T cells, alongside with a trend for increase in CD8^+^ representation and non‐T‐cell populations, the T full differentiation program is preserved into late adulthood [36]. As thymic T‐cell production has been linked even in adults to protection against cancer [28, 29, 31, 49], understanding these dynamics remains of broad clinical interest. Further investigation of the CD8^+^ population in the aged thymus is warranted, as intrathymic memory‐like CD8^+^ T cells may recirculate into the thymus and contribute to organ remodeling and involution [36, 50].

In conclusion, our study delineates discrete developmental transitions in human thymopoiesis occurring sharply from the perinatal through early postnatal period into thymic homeostasis characterized by age‐dependent quantitative and qualitative remodeling of defined thymocyte subsets and provides a valuable resource for understanding human thymic biology.

### 3.1 Data Limitations and Perspectives

The main limitation of this study is the relatively limited number of thymic samples available in certain age groups, particularly in the adult group and frozen cohort. Expanding analyses to larger cohorts will help refine our understanding of thymopoiesis across aging. Findings reported here, which did not include multiple correction, particularly those associated with modest statistical support should be interpreted with caution and considered hypothesis‐generating until validated in independent cohorts. Nevertheless, the inclusion of effect size measurements indicates that several observed differences may be biologically meaningful despite the limited sample size. In addition, our study is cross‐sectional rather than longitudinal, precluding direct tracking of developmental trajectories within individuals. Although high‐dimensional phenotyping provides detailed insights into thymocyte composition and differentiation states, functional validation of TCR signaling strength, effector potential, and lineage commitment was not directly assessed. Future studies, including functional assays, will be important to validate these findings.

Another important limitation of this study is that all thymic samples were obtained from children undergoing corrective surgery for congenital heart disease (CHD). Previous studies have reported reduced thymic output and thymic alterations in children with CHD [51, 52], raising the possibility that some of the features observed in this cohort may be influenced by the underlying pathology or other confounding factors, such as birth weight, prematurity, or steroids administration. Because donor samples were anonymized and detailed clinical information regarding CHD subtype and severity as well as other clinical confounding factors was not available, we could not directly assess the impact of these factors on thymic composition.

## 4 Methods

### 4.1 Isolation of Human Thymocyte Subsets

Pediatric thymus samples were obtained from children and adults undergoing cardiac surgery and used according to and with the approval of the French Ministry of Research (Hôpital Necker, Paris, DC‐2014‐2272, Hôpital Georges Pompidou, Paris, THYMAGE CPP 17 05 29, May 23, 2017). The thymus tissue was mechanically disrupted by cutting the thymic lobes into small pieces and then squeezing the pieces with the plunger of a 5‐mL syringe through a 70‐µm cell strainer to obtain a single cell suspension. Cells were subsequently cryopreserved in liquid nitrogen until further use.

### 4.2 Thymocytes Thawing

Frozen thymocyte suspensions were rapidly thawed in a 37°C water bath, washed with pre‐warmed complete culture medium to remove cryoprotectant, and counted prior to downstream analyses.

### 4.3 Flow Cytometry Analysis

Staining of surface markers was performed in PBS (Gibco) with 0.5% BSA (Gibco) and 2 mM EDTA (Gibco) with titrated antibody to cell ratio, a list of used antibodies is available in Table S1. Intranuclear stainings were performed following the supplier's instructions using FoxP3/TF staining buffer set (eBioscience). Flow cytometric acquisition was performed on the Aurora 5L cytometer (Cytek Biosciences). Flow cytometry data were analyzed using SpectroFlo software (Cytek Biosciences), FlowJo software (TreeStar Inc.) and R.

### 4.4 sjTREC Measure

DNA extraction was performed using the DNeasy Blood and Tissue Kit (Qiagen). Multiplex real time quantification was performed using the 7500 Fast PCR system (Life Technologies) in 96‐well plates loaded with 20 µL containing 8 µL of DNA (0.5 µg of genomic DNA), 10 µL of 2x Takyon Low Rox Probe MM (Eurogentec) and 2 µL of specific primer‐probe mix (Table S2) with the following thermal protocol: 95°C for 10 min and 40 cycles of 95°C for 15 s and 60°C for 60 s. sjTRECs were normalized to 150,000 cells using the Albumin gene quantification and log10‐transformed.

### 4.5 Data Analysis

Flow cytometry data were first processed by gating live CD45^+^ cells, which were then downsampled to ensure balanced representation across samples on FlowJo software (v10.10.1, TreeStar Inc.). FSC were loaded into R (v4.3.2) using the *flowCore* (v2.14.2) package. Fluorescence channels were transformed using the arcsinh function implemented in the *flowVS* (v1.34.0) package to stabilize variance across marker intensities; cofactor values used are available on Table S3. Data quality was assessed and cleaned using *PeacoQC* (v1.12.0), with standard threshold allowing the removal of acquisition anomalies and low‐quality events. Cleaned data from all samples were subsequently integrated into a SingleCellExperiment (SCE) object using the *Catalyst* (v1.26.1) framework for standardized downstream analysis. Clustering of phenotypically similar cells was performed using the FlowSOM algorithm. The resulting metaclusters were visualized using UMAP dimensionality reduction. Statistical analyses were conducted in R. *p* values were not adjusted for multiple testing and are reported for exploratory purposes only. Accordingly, all statistical results should be interpreted as hypothesis‐generating rather than confirmatory.

### 4.6 Sample Inclusion and Cohort Allocation

Human thymic samples were allocated to analyses based on staining and tissue availability. Two partially overlapping cohorts were used: (i) the total cohort, from which we performed sjTREC analysis; (ii) the fresh cohort in which thymocytes were stained immediately after tissue processing; and (iii) a frozen (biobanked) cohort, in which cells were cryopreserved prior to analysis. Differences in cohort size across analyses reflect these cohort‐specific processing constraints. For each analysis, only samples meeting predefined technical quality‐control criteria were included. No sample was excluded based on biological or experimental outcomes. The distribution of samples across analyses is summarized in Figure S8 and Table S5.

## Author Contributions

Camille Kergaravat, Jean‐Marc Doisne, Emmanuel Clave, and Antoine Toubert together designed the study. Camille Kergaravat, Jean‐Marc Doisne, and Clarisse Godard performed data acquisition. Camille Kergaravat performed data analysis. Thymic samples were provided by Agata Cieslak, Vahid Asnafi, and Mourad Amrane. Kamel Benlagha, David Vermijlen, and James P. Di Santo provided guidance. Camille Kergaravat, Jean‐Marc Doisne, Emmanuel Clave, and Antoine Toubert wrote the manuscript. All the authors critically reviewed the manuscript. All the authors revised the manuscript for important intellectual content.

## Conflicts of Interest

The authors declare no conflicts of interest.

## Supporting information




**Supporting File**: eji70257‐sup‐0001‐SuppMat.pdf.

## Data Availability

All code to reproduce analyses and the data necessary to perform the analyses described in the manuscript are available via GitHub at https://github.com/CamilleKergaravat/Thymus_Ressource_Paper.
