# Supplementary material for: Analysis of Human Thymopoiesis Reveals Rapid Postnatal Remodeling of Thymocytes Within the First 3 Months of Life
Source: Eur J Immunol. 2026 Aug 18;56(8):e70257. doi: 10.1002/eji.70257 (PMC13482440; doi:10.1002/eji.70257)
Supplement: Supplementary file 1 [file EJI-56-e70257-s001.pdf]

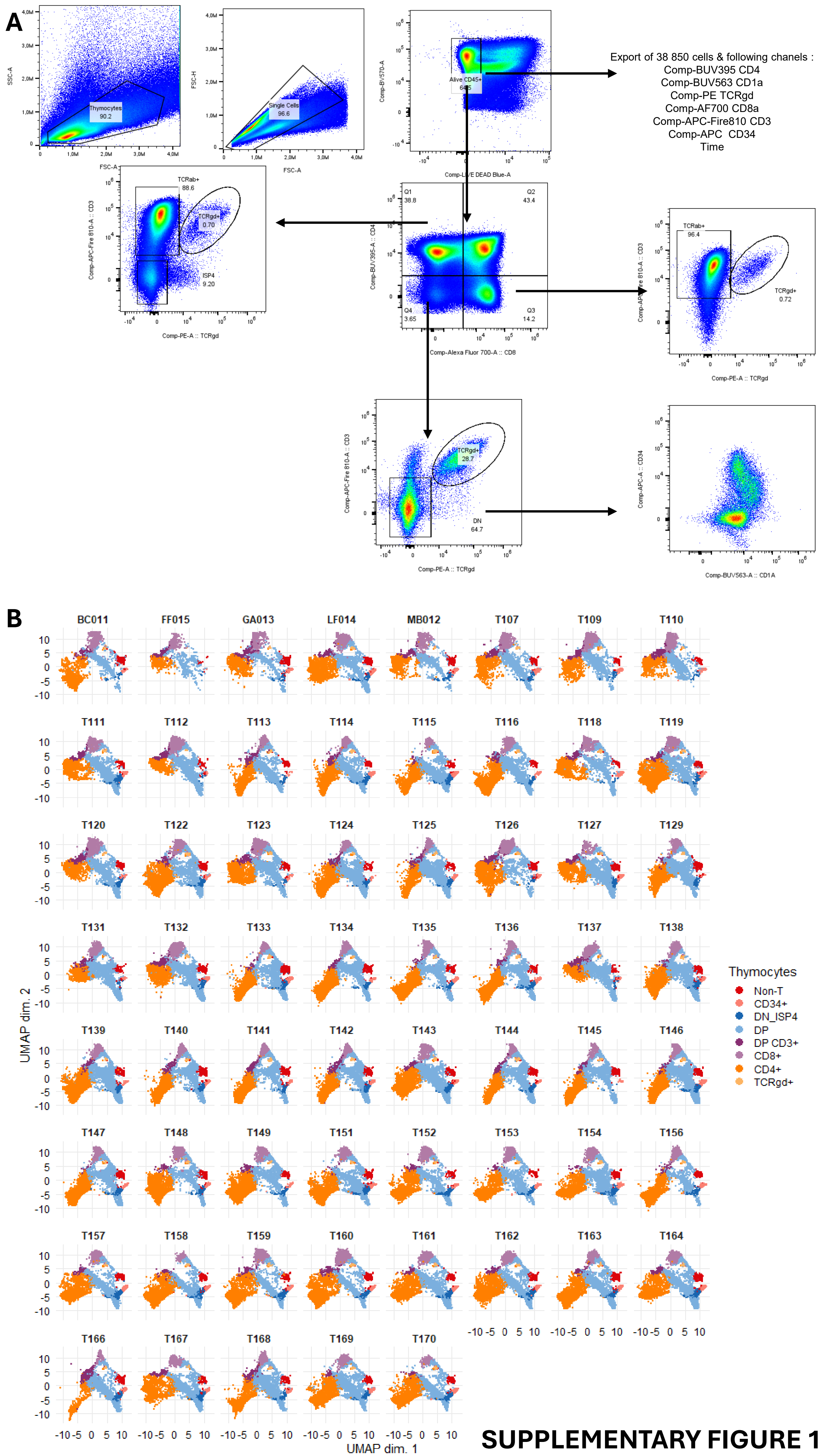

SUPPLEMENTARY FIGURE 1

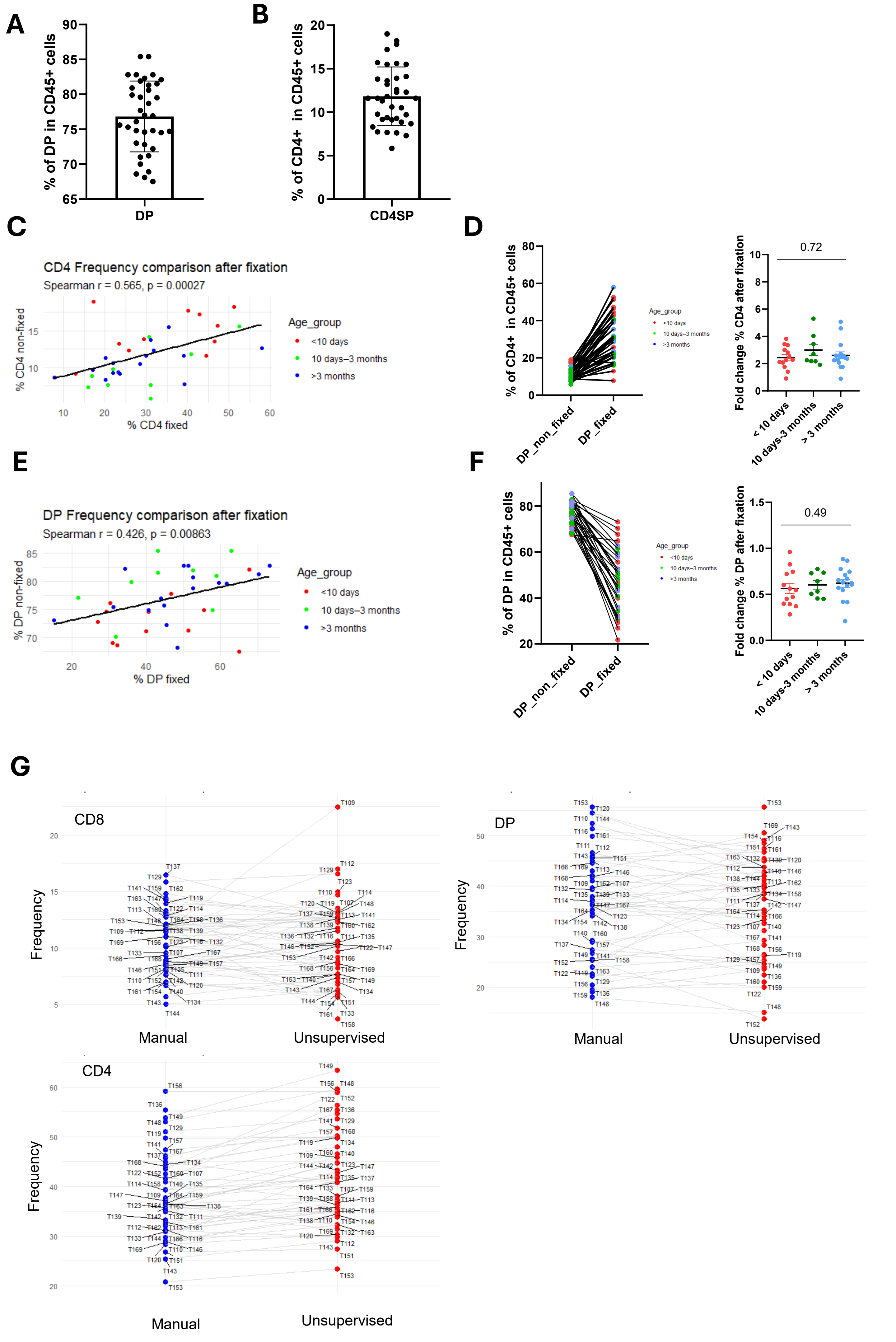

SUPPLEMENTARY FIGURE 2

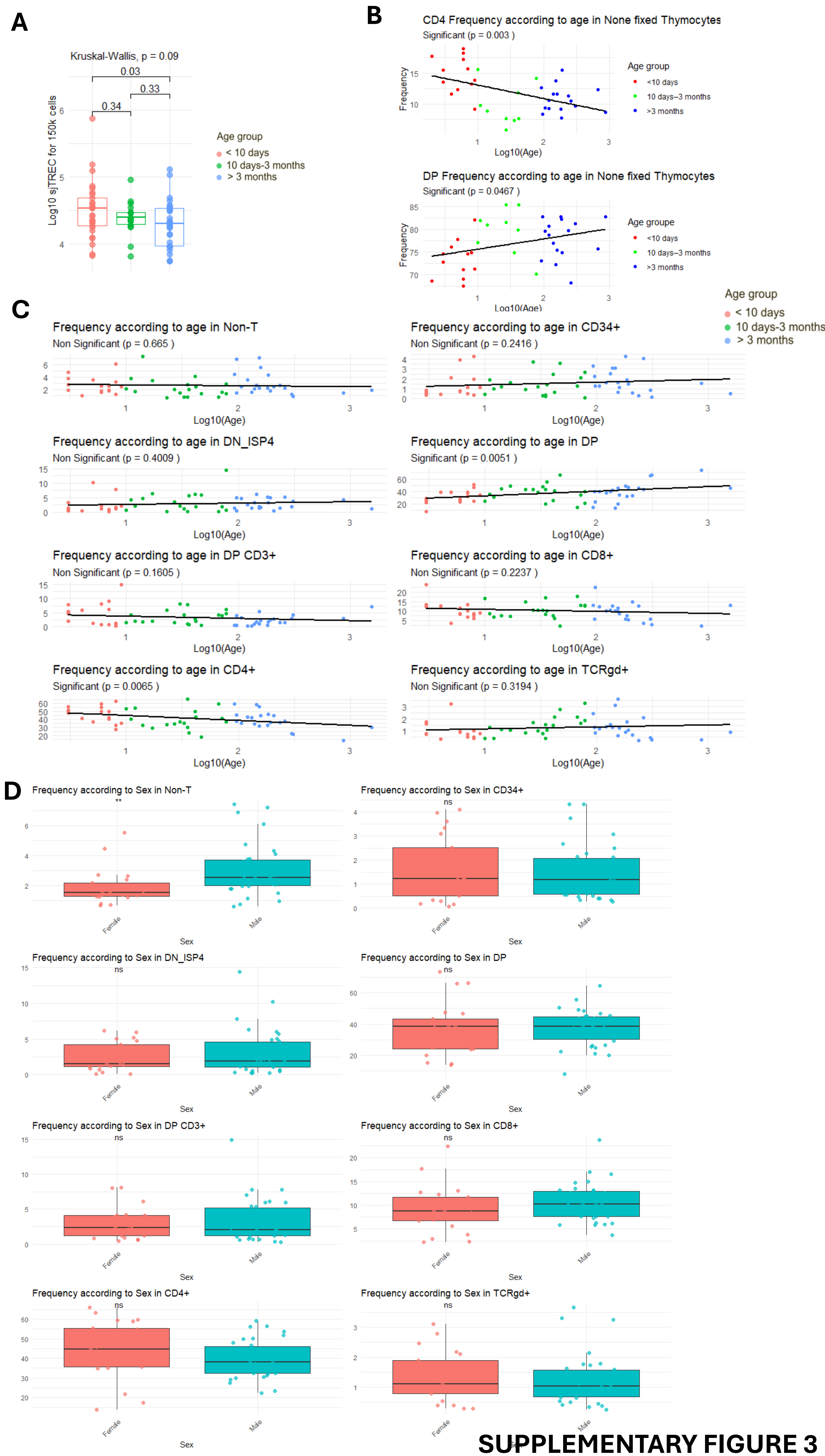

SUPPLEMENTARY FIGURE 3

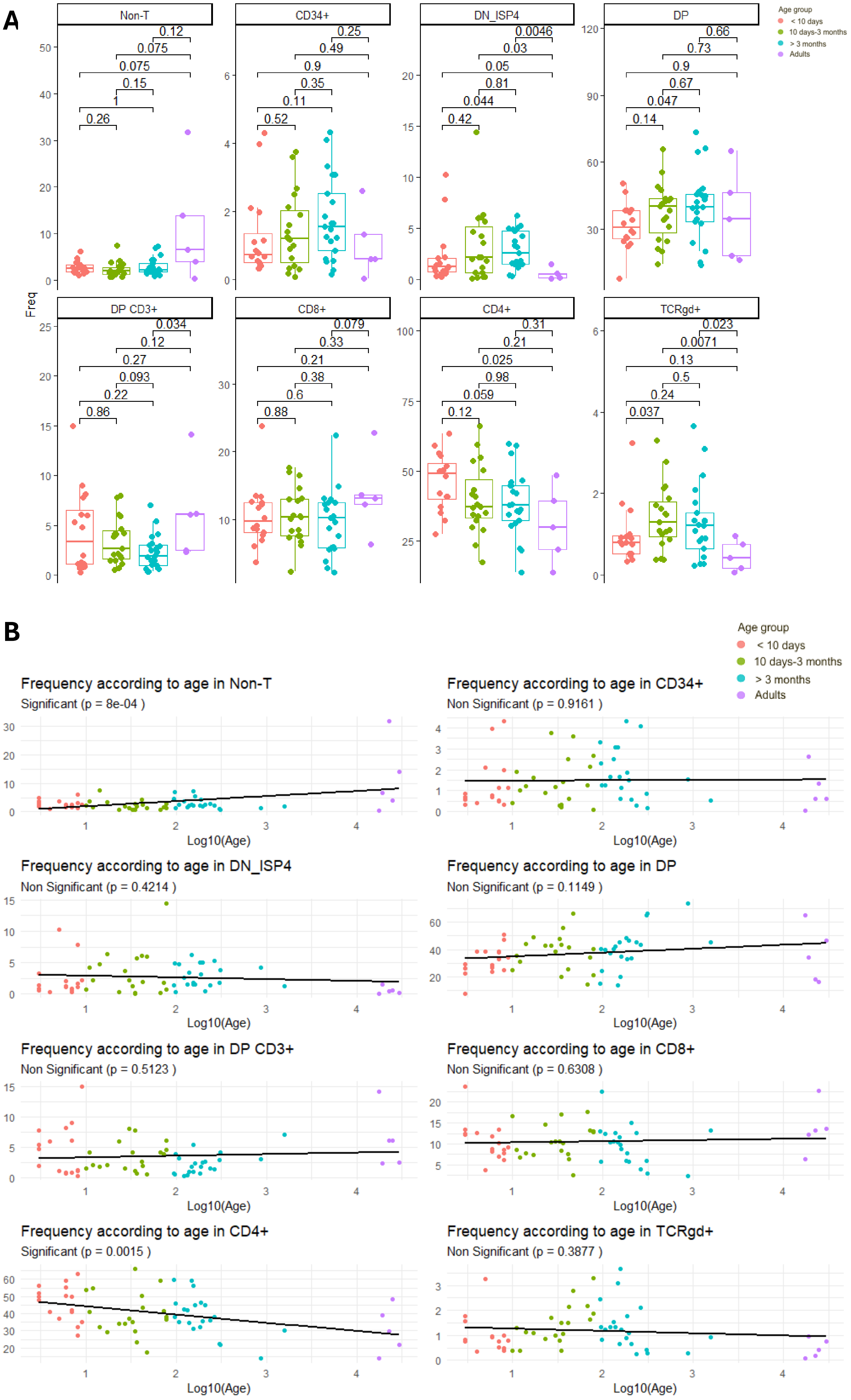

SUPPLEMENTARY FIGURE 4

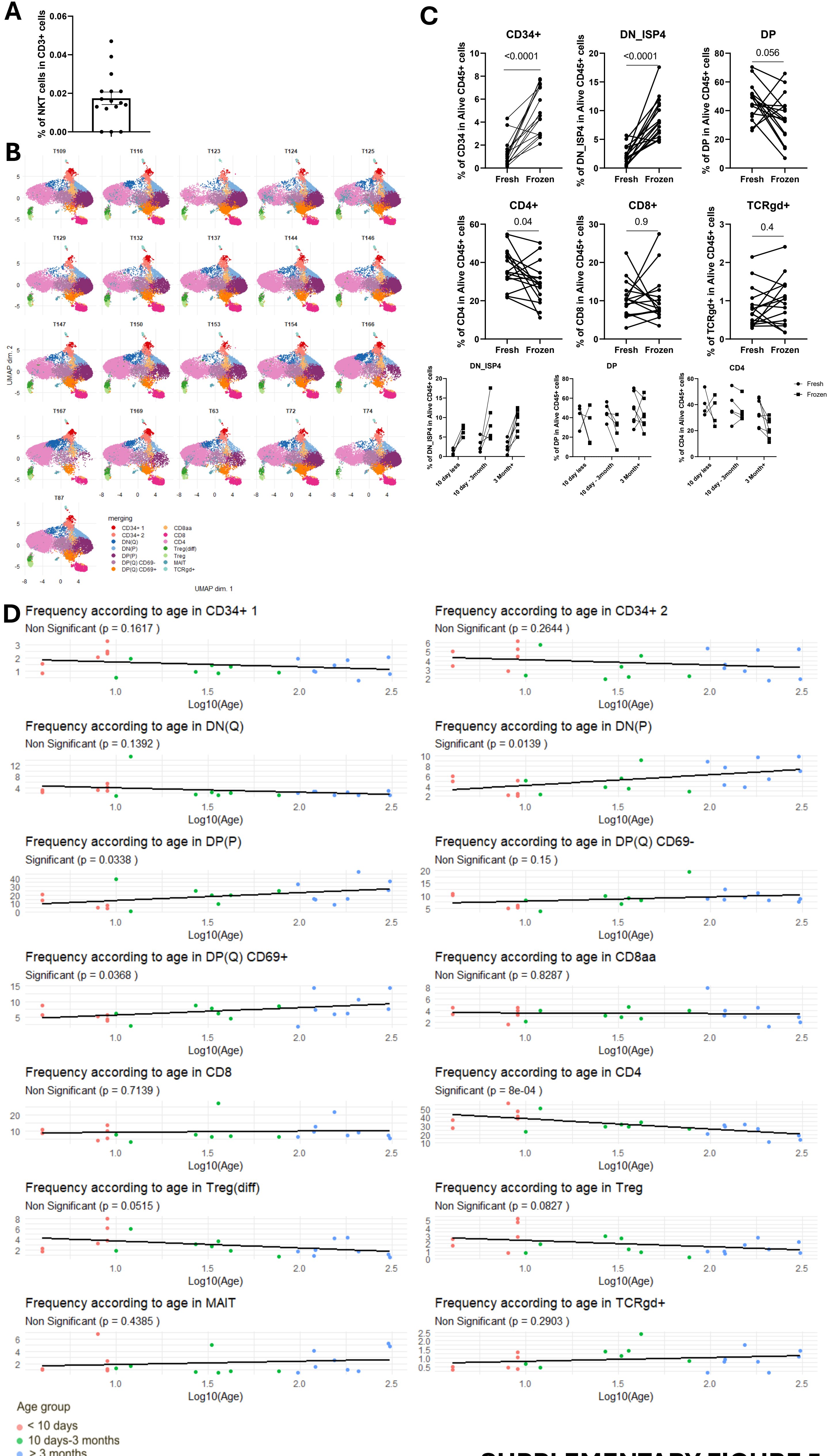

SUPPLEMENTARY FIGURE 5

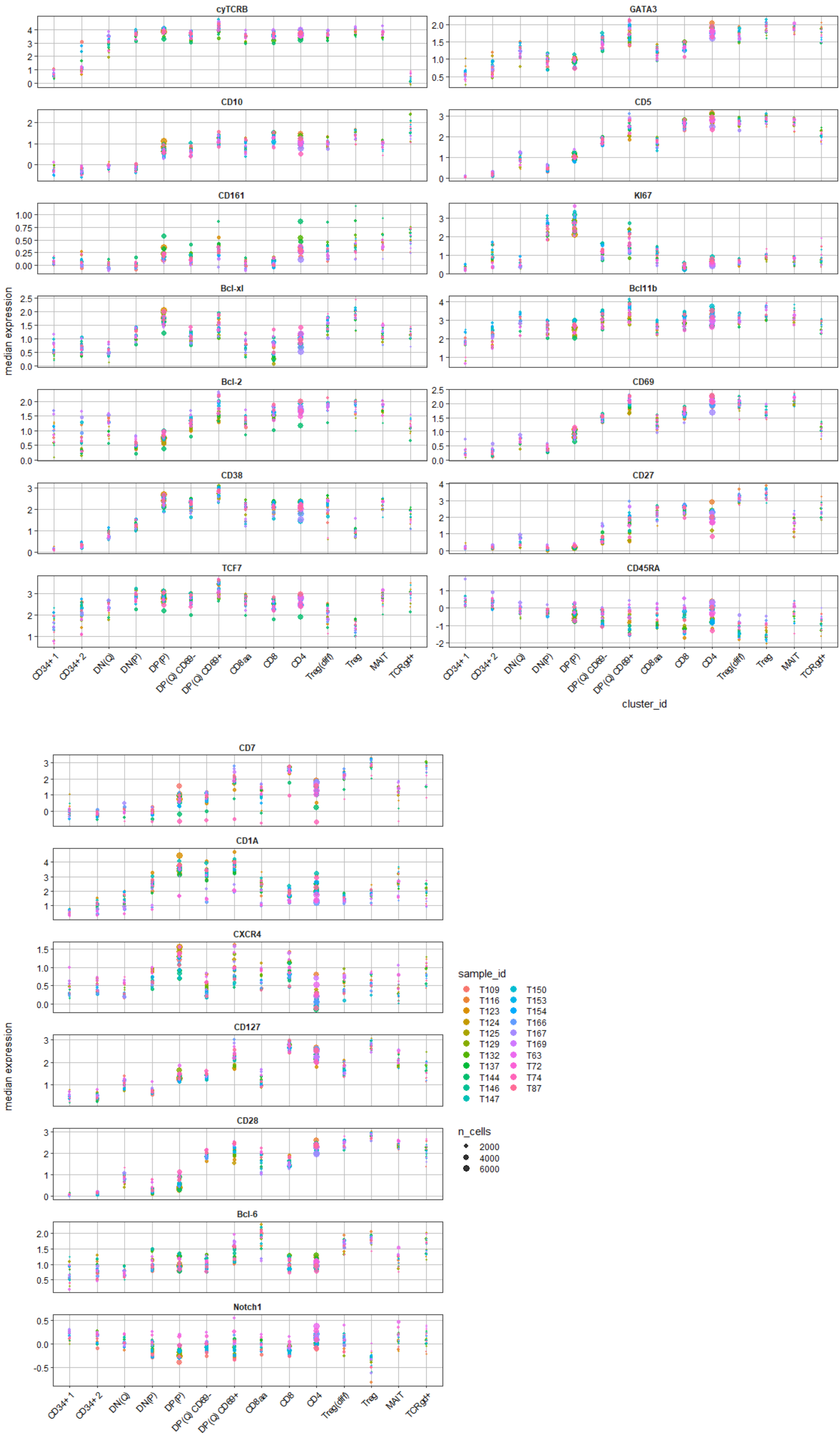

SUPPLEMENTARY FIGURE 6

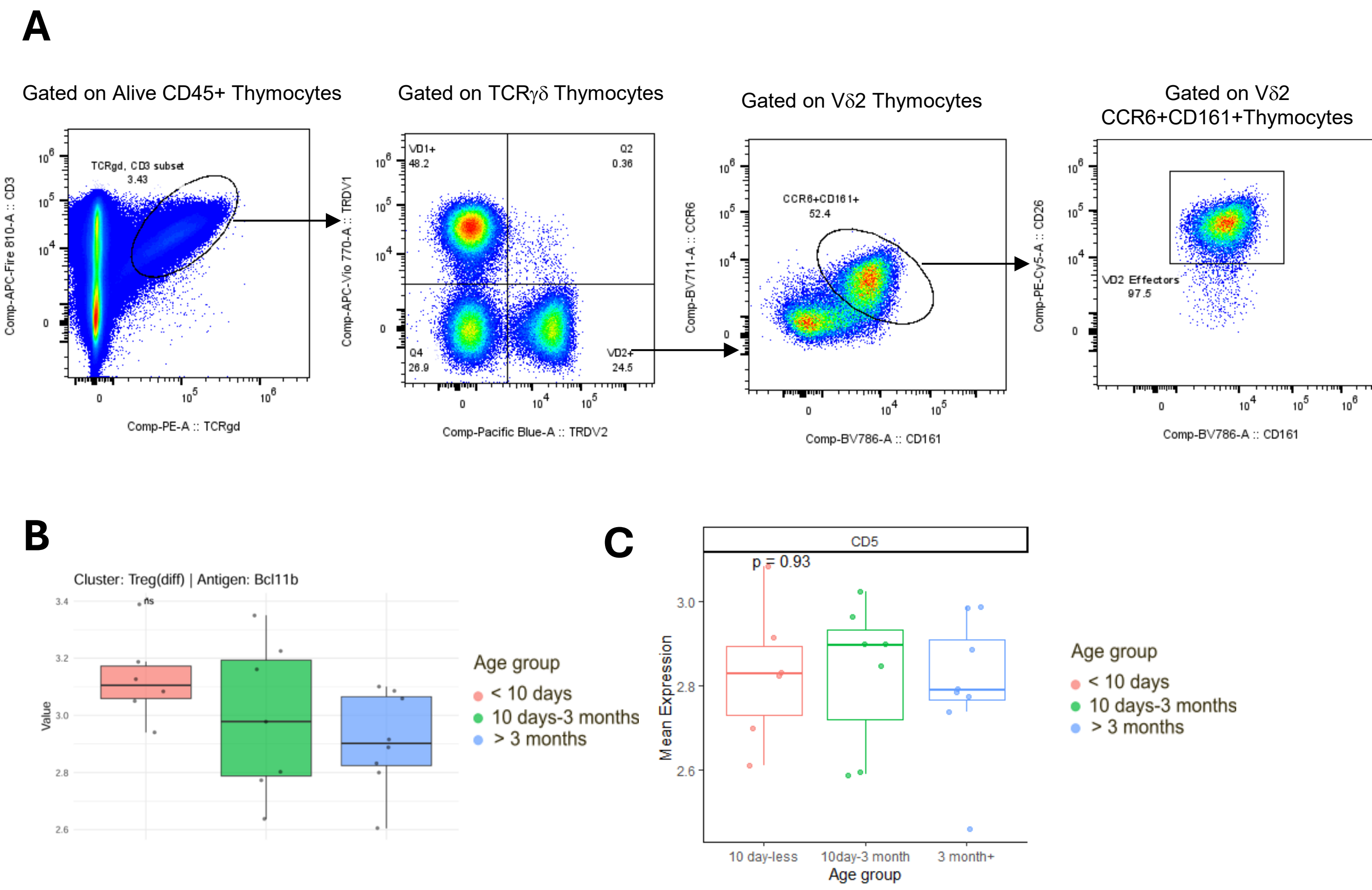

SUPPLEMENTARY FIGURE 7

A

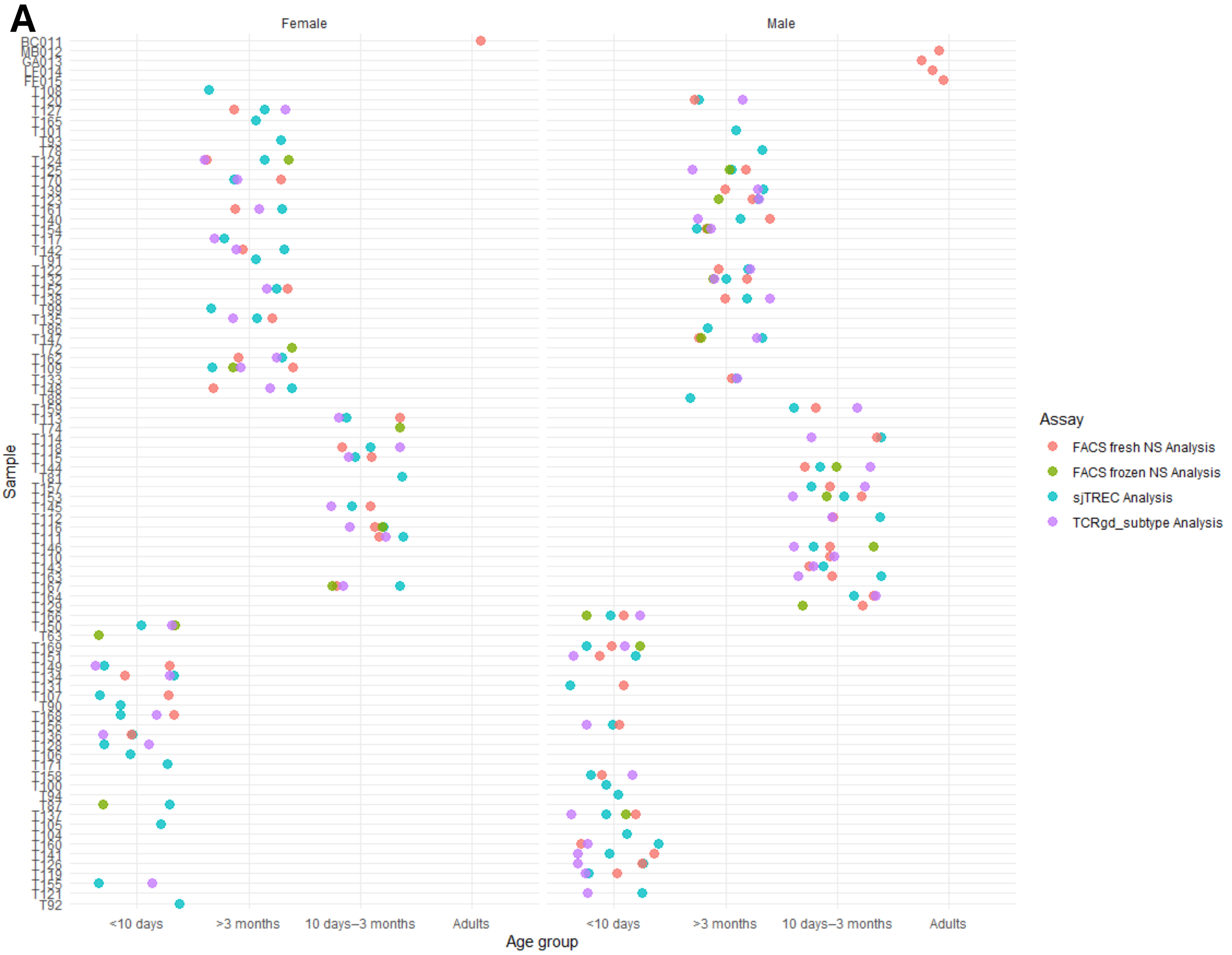

B

Analysis	< 10 days	10 days-3 months	> 3 months	Rationale for reduced n	Figure
sjTREC	29	18	31	Full thymic cohort & qPCR QC	Figure 2A
FACS Fresh staining NS Analysis	16	19	21	Availibility of fresh staining & NS QC	Figure 1, Figure 2B-F, Figure 5B
FACS Frozen staining NS Analysis	6	7	8	Reduced sample inclusion	Figure 3-5
FACS Fresh staining TCRgd Analysis	18	18	21	Availibility of fresh staining & TCRgd staining QC	Figure 5 C-D

SUPPLEMENTARY FIGURE 8

Supplementary Figure 1. Human Thymocyte Composition in Fresh Thymic Tissue: the Unsupervised Analysis Approach

(A) Schematic representation of the gating strategy, downsampling procedure, export of flow cytometry channels used for FCS file processing and manual gating scheme of main thymocytes population.

(B) UMAP projection of human thymocytes colored according to annotated clusters for each individual sample.

Supplementary Figure 2. Human thymocyte composition in fresh thymic tissue: evaluation of fixation effects and validation of the unsupervised approach.

(A) Boxplots showing the frequency of DP (double-positive) thymocytes among live $CD45^+$ thymocytes in unfixed samples (n = 37).

(B) Boxplots showing the frequency of $CD4^+$ thymocytes among live $CD45^+$ thymocytes in unfixed samples (n = 37).

(C) Correlation between frequencies of $CD4^+$ thymocytes among live $CD45^+$ thymocytes measured in fixed and unfixed samples (n = 37). The R value corresponds to the Spearman correlation coefficient, and the p value indicates the significance of the correlation.

(D) Left: Comparison of $CD4^+$ thymocyte frequencies among $CD45^+$ thymocytes measured in fixed and unfixed samples across age groups (<10 days, n = 13; 10 days–3 months, n = 8; >3 months, n = 16). Right: Fold change in $CD4^+$ thymocyte frequencies between fixed and unfixed samples across age groups (<10 days, n = 13; 10 days–3 months, n = 8; >3 months, n = 16) P value is calculated using the Kruskal–Wallis test.

(E) Correlation between frequencies of DP thymocytes among $CD45^+$ thymocytes measured in fixed and unfixed samples (n = 37). The R value corresponds to the Spearman correlation coefficient, and the p value indicates the significance of the correlation.

(F) Left: Comparison of DP thymocyte frequencies among $CD45^+$ thymocytes measured in fixed and unfixed samples across age groups (<10 days, n = 13; 10 days–3 months, n = 8; >3 months, n = 16). Right: Fold change in DP thymocyte frequencies between fixed and unfixed samples across age groups (<10 days, n = 13; 10 days–3 months, n = 8; >3 months, n = 16). P values were calculated using the Kruskal–Wallis test.

(G) Comparison of population frequencies obtained by manual gating and unsupervised analysis for $CD8^+$, DP (double-positive), and $CD4^+$ in fresh fixed thymocyte populations.

Supplementary Figure 3. Early-Life Age- and Sex-Related Changes in Pediatric Thymocyte Composition and changes in sjTRECs levels

(A) sjTREC levels according to age. Boxplots showing log10-transformed sjTREC levels across age groups (<10 days, n = 29; 10 days–3 months, n = 18; >3 months, n = 31). P values correspond to pairwise comparisons between age groups and were calculated using two-sided Wilcoxon rank-sum tests and global Kruskal-Wallis test.

(B) Linear regression analysis of $CD4^+$ (top) and DP (double-positive; bottom) thymocyte frequencies as a function of log10-transformed age in unfixed fresh thymocytes (<10 days, n = 13; 10 days–3 months, n = 8; >3 months, n = 16). P values correspond to the significance of the regression slope.

(C) Linear regression analysis of thymocyte subpopulation frequencies as a function of log10-transformed age in fresh fixed samples (<10 days, n = 16; 10 days–3 months, n = 19; >3 months, n = 21). P values correspond to the significance of the regression slope.

(D) Boxplots showing thymocyte subpopulation frequencies according to sex (female, n = 22; male, n = 34). P values were calculated using the Wilcoxon rank-sum test.

Supplementary Figure 4. Age-Related Changes in Thymocyte Composition

(A) Boxplots showing thymocyte subpopulation frequencies across age groups (<10 days, n = 16; 10 days–3 months, n = 19; >3 months, n = 21; Adults, n = 5). P values correspond to pairwise comparisons between age groups and were calculated using two-sided Wilcoxon rank-sum tests.

(B) Linear regression analysis of thymocyte subpopulation frequencies as a function of log10-transformed age (<10 days, n = 16; 10 days–3 months, n = 19; >3 months, n = 21; Adults, n = 5). P values correspond to the significance of the regression slope.

Supplementary Figure 5. Validation of the Biobanked Thymocyte Profiling Approach

(A) Boxplots showing the frequency of NKT cells among $CD3^+$ thymocytes (n = 21).

(B) UMAP projection of human thymocytes colored according to annotated clusters for each individual sample.

(C) Top, comparison of population frequencies recovered from frozen and fresh samples within the major thymocyte populations (n=16). Bottom, comparison of population frequencies recovered from frozen and fresh samples within the DN_ISP4, DP, and CD4 clusters across age groups (<10 days, n = 4; 10 days–3 months, n = 5; >3 months, n = 7). P values were calculated using the Wilcoxon rank-sum test.

(D) Linear regression analysis of thymocyte subpopulation frequencies in frozen samples as a function of log10-transformed age (<10 days, n = 6; 10 days–3 months, n = 7; >3 months, n = 8). P values correspond to the significance of the regression slope.

Supplementary Figure 6. Distribution of marker expression across samples and clusters in the frozen cohort.

Supplementary Figure 7. Age-Related Changes Within Unconventional T-Cell Lineages
(A) Schematic representation of the gating strategy used to identify Vδ2 effector TCRγδ cells.
(B) Boxplots showing Bcl11b median expression within the Treg(diff) cluster across age groups (<10 days, n = 6; 10 days–3 months, n = 7; >3 months, n = 8). P value was calculated using one-way ANOVA.
(C) Boxplots showing CD5 median expression within the Treg cluster across age groups (<10 days, n = 6; 10 days–3 months, n = 7; >3 months, n = 8). P value was calculated using one-way ANOVA.

Supplementary Figure 8. Summary of Sample Inclusion Across Analyses
(A) Schematic overview of all thymic samples included in each analysis, stratified by age group (<10 days, 10 days–3 months, >3 months, Adults) and sex.
(B) Table summarizing donor inclusion for each figure and analysis and reasons for reduced sample size.

Supplementary Table 1 **List of used antobodies**

Surface markers

Fluorochrome	Antibody	Clone	Panel
Biot	CD14	REA599	Frozen
Biot	CD19	REA675	Frozen
Biot	CD20	2H7	Frozen
Biot	CD66abce	REA1230	Frozen
Biot	CD123	REA918	Frozen
Biot	CD203c	REA826	Frozen
Biot	CD303	REA693	Frozen
Biot	FceRIa	AER-37(CRA-1)	Frozen
FVD eFluor 455UV	L/D		Frozen
LIVE/DEAD Blue UV	L/D		Fresh
BUV395	CD4	RP4-T4	Fresh
BUV496	CD7	M-T701	Frozen
BUV563	CD1a	HI149	Fresh
BUV563	CD10	HI10a	Frozen
BUV615	CD5	UCHT2	Frozen
BUV661	TCRgd	11F2	Frozen
BUV737	CD127	HIL-7R-M21	Fresh
BUV805	CD34	581	Frozen
BUV805	CD38	HB-7	Fresh
BV421	CD3	UCHT1	Frozen
BV480	CD8b	2ST8.5H7	Frozen
BV480	CD16	3G8	Fresh
BV570	CD45	HI30	Fresh
StarBright Violet 570	CD45RA	F8-11-13	Frozen
BV605	CD1a	HI149	Frozen
BV650	CD161	HP-3G10	Frozen
BV650	CD197	G043H7	Fresh
BV711	CD196	11A9	Fresh
BV750	Va7.2	OF-5A12	Frozen
BV785	CXCR4	12G5	Frozen
BV786	CD161	HP-3G10	Fresh
FITC	TCRab	IP26	Fresh
RB545	CD45	HI30	Frozen
StarBright Blue 580	CD8a	LT8	Frozen
RB613	CD38	HB7	Frozen
RB670	CD4	SK3	Frozen
BB700	CD194	1G1	Fresh
RB744	CD27	M-T271	Frozen
PE	TCRgd	5A6.E91	Fresh
PE-CF594	PD1	EH12	Fresh
PE-Fire640	CD69	FN50	Frozen
PE-Cy5	CD26	BA5b	Fresh
PE-AF700	CD25	CD25-3G10	Frozen
PE-Cy7	CD127	eBioRDR5	Frozen
PE-Cy7	CD94	DX22	Fresh

**PE-Fire810**	**CD28**	CD28.2	Frozen
**APC**	**CD34**	8G12	Fresh
**AF700**	**CD8**	SK1	Fresh
**APC-Vio770**	**iNKT**	REA1054	Frozen
**APC-Vio770**	**TCRVD1**	REA173	Fresh
**APC-Fire 810**	**CD3**	SK7	Fresh
**APC-Fire810**	**TCRab**	IP26	Frozen

Intracellular markers

Fluorochrome	Antibody	Clone	Panel
**BUV395**	**GATA-3**	L50-823	Frozen
**BV421**	**Ki-67**	B56	Fresh
**BV711**	**Ki-67**	B56	Frozen
**FITC**	**Bcl-xL**	7B2.5	Frozen
**RB705**	**TCF-7/TCF-1**	S33-966	Frozen
**RB780**	**Bcl-6**	K112-91	Frozen
**PE**	**Bcl11b**	W17189A	Frozen
**PE-CF594**	**Bcl-2**	Bcl-2/100	Frozen
**RY703**	**FoxP3**	259D/C7	Frozen
**APC**	**cyTCRB**	8A3	Frozen
**R718**	**Notch1**	MHN1-519	Frozen

Supplementary Table 2 **List of used primers**

Assay	Probe	F- Primer	R- Primer
sjTREC	*FAM* -ACACCTCTGGTTTTTGTAAAGGTG-*EclipseDQ*	CACATCCCTTTCAACCATGCT	GCCAGCTGCAGGGTTTAGG
Albumin	*FAM* -CCTGTCATGCCCACACAAATCTCTCC-*TAMRA*	GCTGTCATCTCTTGTGGGCTGT	ACTCATGGGAGCTGCTGGTTC

Supplementary Table 3 List of used cofactors

Fresh Panel

Fluorochrom	cofactors
FJComp-APC-A	1982
FJComp-APC-Fire 810-A	1609,74128
FJComp-Alexa Fluor 700-A	878,066302
FJComp-BUV395-A	766,066518
FJComp-BUV563-A	856,092414
FJComp-PE-A	12810,1085

Frozen Panel

Fluorochrom	cofactors
FJComp-APC-A	755,065651
FJComp-APC-Fire 810-A	1227,72338
FJComp-APC-Vio 770-A	2000
FJComp-BUV395-A	961,314386
FJComp-BUV496-A	2261,20995
FJComp-BUV563-A	1923,4798
FJComp-BUV615-A	4183,1078
FJComp-BUV661-A	567,072146
FJComp-BUV805-A	670
FJComp-BV421-A	4607,13713
FJComp-BV480-A	1194,7062
FJComp-BV605-A	1532,756
FJComp-BV650-A	2000
FJComp-BV711-A	1969,0134
FJComp-BV750-A	1629,28717
FJComp-BV786-A	2500
FJComp-FITC-A	1478,65106
FJComp-PE-A	617,5467
FJComp-PE-Alexa Fluor 70	1978,55918
FJComp-PE-Cy7-A	1000,5164
FJComp-PE-Dazzle594-A	8676,09535
FJComp-PE-Fire 640-A	4937,436
FJComp-PE-Fire810-A	1942,00291
FJComp-RB613-A	17187,7872
FJComp-RB670-A	302,739568
FJComp-RB744-A	3051
FJComp-RB780-A	1541,46191
FJComp-RY703-A	3557,5285
FJComp-RealBlue 705-A	2003,90048
FJComp-SBB580-A	1048,42128
FJComp-StarBright Violet	1266,54262
FJComp-r718-A	1000

Supplementary Table 4- List of p values and effect size for the frozen cohort

Figure	Variable	p_val	Effect size
4A	TCF7	0.05	R^2 = 0.17
4A	Bcl-6	0.004	R^2 = 0.34
4B	CD1a	0.04	0.28
4B	Bcl-2	0.02	0.32
4B	Ki-67	0.007	R^2 = 0.32
4C	CD5	0.0005	0.47
4C	CD69	0.01	0.35
4C	CD28	0.01	0.37
4D	CD5	0.01	R^2 = 0.25
4E	CD127	0.001	0.19
4E	CD45RA	0.02	0.11
5A	Treg	0.048	0.28
5E	Bcl11b	0.04	0.29
5E	Ki-67	0.04	0.28
5F	CD10	0.018	0.36
5F	CD161	0.09	0.22
5F	Bcl-6	0.02	0.32

Supplementary Table 5- Summary of cohort composition, donors information and inclusion in each analysis

Sample_id	Sex	Age (days)	age_group	sjTREC Analysis	FACS fresh NS Analysis	FACS frozen NS Analysis	TCRgd_subtype Analysis
T92	Female	1	<10 days	included			
T121	Male	2	<10 days	included			included
T155	Female	2	<10 days	included			included
T119	Male	3	<10 days	included	included		included
T126	Male	3	<10 days	included	included		included
T141	Male	3	<10 days	included	included		included
T160	Male	3	<10 days	included	included		included
T137	Male	4	<10 days	included	included	included	included
T104	Male	4	<10 days	included			
T105	Female	4	<10 days	included			
T87	Female	4	<10 days	included		included	
T94	Male	4	<10 days	included			
T158	Male	5	<10 days	included	included		included
T100	Male	5	<10 days	included			
T171	Female	5	<10 days	included			
T128	Female	6	<10 days	included			included
T136	Female	6	<10 days	included	included		included
T156	Male	6	<10 days	included	included		included
T168	Female	6	<10 days	included	included		included
T106	Female	6	<10 days	included			
T90	Female	6	<10 days	included			
T134	Female	7	<10 days	included	included		included
T107	Female	7	<10 days	included	included		
T131	Male	7	<10 days	included	included		
T149	Female	8	<10 days	included	included		included
T151	Male	8	<10 days	included	included		included
T169	Male	8	<10 days	included	included	included	included
T63	Female	8	<10 days			included	
T150	Female	9	<10 days	included		included	included
T166	Male	9	<10 days	included	included	included	included
T129	Male	10	10 days–3 months		included	included	
T164	Male	11	10 days–3 months	included	included		included
T167	Female	12	10 days–3 months	included	included	included	included
T163	Male	14	10 days–3 months	included	included		included
T143	Male	17	10 days–3 months	included	included		included
T110	Male	23	10 days–3 months		included		included
T146	Male	27	10 days–3 months	included	included	included	included
T111	Female	30	10 days–3 months	included	included		included
T116	Female	33	10 days–3 months	included	included	included	included
T112	Male	35	10 days–3 months	included	included		included
T145	Female	35	10 days–3 months	included	included		included
T153	Male	36	10 days–3 months	included	included	included	included
T157	Male	41	10 days–3 months	included	included		included
T81	Female	41	10 days–3 months	included			
T144	Male	42	10 days–3 months	included	included	included	included
T115	Female	47	10 days–3 months	included	included		included
T118	Female	67	10 days–3 months	included	included		included
T114	Male	72	10 days–3 months	included	included		included
T74	Female	77	10 days–3 months			included	
T113	Female	78	10 days–3 months	included	included		included
T159	Male	78	10 days–3 months	included	included		included
T88	Male	92	>3 months	included			
T148	Female	93	>3 months	included	included		included
T133	Male	96	>3 months	included	included		included
T109	Female	97	>3 months	included	included	included	included
T162	Female	106	>3 months	included	included		included
T72	Female	119	>3 months			included	
T147	Male	121	>3 months	included	included	included	included
T86	Male	122	>3 months	included			
T135	Female	132	>3 months	included	included		included
T99	Female	132	>3 months	included			
T138	Male	139	>3 months	included	included		included
T152	Female	146	>3 months	included	included		included
T132	Male	154	>3 months	included	included	included	
T122	Male	155	>3 months	included	included		included
T91	Female	156	>3 months	included			
T142	Female	159	>3 months	included	included		included
T117	Female	180	>3 months	included			included
T154	Male	182	>3 months	included	included	included	included
T140	Male	186	>3 months	included	included		included
T161	Female	193	>3 months	included	included		included
T123	Male	208	>3 months	included	included	included	included
T139	Male	237	>3 months	included	included		included
T170	Female	262	>3 months	included	included		included
T125	Male	302	>3 months	included	included	included	included
T124	Female	309	>3 months	included	included	included	included
T78	Male	341	>3 months	included			
T93	Female	540	>3 months	included			
T101	Male	612	>3 months	included			
T165	Female	671	>3 months	included			
T127	Female	869	>3 months	included	included		included
T120	Male	1567	>3 months	included	included		included
T108	Female	1742	>3 months	included			
FF015	Male	17520	Adults		included		
LF014	Male	18980	Adults		included		
GA013	Male	22630	Adults		included		
MB012	Male	24820	Adults		included		
BC011	Female	29565	Adults		included		
